# SAXS-guided Enhanced Unbiased Sampling for Structure Determination of Proteins and Complexes

**DOI:** 10.1038/s41598-018-36090-z

**Published:** 2018-12-10

**Authors:** Chuankai Zhao, Diwakar Shukla

**Affiliations:** 1 Department of Chemical and Biomolecular Engineering, University of Illinois at Urbana-Champaign, Urbana, IL 61801 United States; 2 Department of Plant Biology, University of Illinois at Urbana-Champaign, Urbana, IL 61801 United States; 3 Center for Biophysics and Quantitative Biology, University of Illinois at Urbana-Champaign, Urbana, IL 61801 United States; 4 National Center for Supercomputing Applications, University of Illinois at Urbana-Champaign, Urbana, IL 61801 United States

## Abstract

Molecular simulations can be utilized to predict protein structure ensembles and dynamics, though sufficient sampling of molecular ensembles and identification of key biologically relevant conformations remains challenging. Low-resolution experimental techniques provide valuable structural information on biomolecule at near-native conditions, which are often combined with molecular simulations to determine and refine protein structural ensembles. In this study, we demonstrate how small angle x-ray scattering (SAXS) information can be incorporated in Markov state model-based adaptive sampling strategy to enhance time efficiency of unbiased MD simulations and identify functionally relevant conformations of proteins and complexes. Our results show that using SAXS data combined with additional information, such as thermodynamics and distance restraints, we are able to distinguish otherwise degenerate structures due to the inherent ambiguity of SAXS pattern. We further demonstrate that adaptive sampling guided by SAXS and hybrid information can significantly reduce the computation time required to discover target structures. Overall, our findings demonstrate the potential of this hybrid approach in predicting near-native structures of proteins and complexes. Other low-resolution experimental information can be incorporated in a similar manner to collectively enhance unbiased sampling and improve the accuracy of structure prediction from simulation.

## Introduction

Proteins fold into precise three-dimensional structures to carry out essential cellular functions such as enzyme catalysis^1–5^ and signaling^6–12^. To understand protein structure-function relationships, it is crucial to obtain knowledge of not only key biologically relevant functional conformations but also kinetic pathways associated with the conformational change process. Although X-ray crystallography and nuclear magnetic resonance (NMR) techniques can provide high-resolution protein structures, it is often difficult to capture all conformation states of proteins in solution. Complementarily, low-resolution experimental techniques, such as small angle X-ray scattering (SAXS)^13^, single molecule fluorescence resonance energy transfer (smFRET)^14^ and double electron-electron resonance (DEER)^15^ can be utilized to gain insights into the protein conformational states or dynamics in solution. However, due to the low information content of experimental data, these techniques alone are not sufficient to obtain high-resolution protein structures. Instead, additional physical or structural information is required to interpret the information encoded by low-resolution experimental data and prevent overfitting during structure determination and refinement.

Molecular dynamics (MD) simulation is a powerful tool to complement low-resolution experimental data to predict protein structures and ensembles, as well as thermodynamics and kinetics associated with protein function^16,17^. One popular way is to utilize coarse grained MD simulation to generate structural ensemble and then refine the ensemble against experimental data^18–21^. However, coarse grained simulations might not be accurate enough to represent structural ensemble in atomic detail. To improve the predictive accuracy, a recent study utilizes extensive all atom MD simulations to generate a strong prior structural ensemble and incorporates experimental information in a statistical approach to refine the ensemble against experimental data by perturbing the weight of each state in the kinetic model^22^. This method can likely generate the true ensemble under sufficient sampling of molecular ensembles. However, this approach involves *post-hoc* validation of the conformational ensembles based on the extensive simulation data obtained from unbiased MD simulations. It is computationally demanding to generate extensive simulation data for biological processes where conformational dynamics takes place over a millisecond or even longer timescales (i.e. protein folding) or where large biomolecule sizes can greatly hamper the computation speed (i.e. protein-protein association).

To address some of these limitations, low-resolution experimental data, which offers a direct observable to compare simulation with experiment, can be combined with MD sampling process to accelerate discovery of functionally relevant structures of proteins. To achieve this, experimental data is often incorporated as constraints in enhanced sampling algorithms, either through modifying potential functions^23–27^ or defining reaction coordinates^28–30^, to bias simulations and drive molecules of interests towards conformational states that are consistent with experimental data. Although these methods have improved computational efficiency and succeeded in predicting functional conformations of proteins, they may sacrifice kinetic information for accurate thermodynamics.

In this study, we aim to explore how unbiased MD simulations and low-resolution experimental data could be integrated to obtain accurate conformational ensembles of proteins, while at the same time reducing computational costs. In particular, we explore to quantify the advantage of incorporating low-resolution experimental data in conformational sampling algorithm by evaluating the effectiveness of this approach in terms of both structure prediction accuracy and sampling efficiency. Here, SAXS is used as the source of low-resolution structural information due to its wide spread use in structural characterization of both structured and intrinsically disordered biomolecules in solution, especially for complexes^13,31,32^. SAXS data is presented as a one dimensional scattering curve determined from the spherical averaging of random orientations that a biomolecule can adopt in solution. It remains elusive how the information is distributed over the range of scattering curve, however usually experimental SAXS profiles do not contain more than 10–30 independent points^17,33^. Low-resolution shapes of biomolecules can be reconstructed using SAXS data, and structural features such as radius of gyration (R_*g*_) and maximum diameter (D_*max*_) of biomolecules can be extracted by fitting SAXS profile. The low information content leads to the inherent ambiguity of SAXS data that biomolecules with different shape topologies and internal structures can display identical SAXS profiles^31,33,34^. Thanks to the recent advances in SAXS data collection with reduced noises and errors, as well as accurate prediction of SAXS profile from structural models, it becomes possible to harvest the structural information encoded in SAXS data^17,35^.

We present an approach that adopts the SAXS information as a seed selection criteria for Markov state model (MSM)-based adaptive sampling^36^ in unbiased MD simulations to enhance the sampling of protein dynamics and identify near-native conformational states of proteins. As compared to experimental-guided enhanced sampling algorithms, this approach does not introduce changes to MD potential functions, thereby providing more accurate description of equilibrium protein dynamics. Furthermore, different types of structural information can be incorporated at the same time to collectively enhance the sampling of protein dynamics. In this work, we demonstrate this method in the study of protein folding and protein-protein association. Especially, considering the under-determined nature of SAXS data, we aim to explore what additional information might be needed to combine with SAXS to identify native states of proteins and complexes from MD simulations and to better enhance MD sampling efficiency.

To first demonstrate how SAXS and SAXS-based hybrid information can be used to both predict the native structure of single domain protein and reduce the computation time to discover the target structure, we study the foldings of three proteins, including HP35 double norleucine mutant domain (35 residues)^37^, protein G (56 residues)^38^ and *α*3D (73 residues)^38^. Markov state models (MSMs)^39,40^ are constructed using the previously published extensive MD simulation datasets. We show that the combination of SAXS and thermodynamic information estimated from the MSMs is sufficient to clearly differentiate structures and recognize the folded structure for the three small single domain proteins. By performing kinetic Monte Carlo sampling^41^ using different sampling protocols on the MSMs, we show that SAXS-guided adaptive sampling strategy greatly reduces the simulation cost of reaching the target structure as compared to other sampling methods. We also demonstrate that distance restraints inferred from intramolecular evolutionary couplings (ECs) can be combined with SAXS to further improve the prediction accuracy and sampling efficiency.

Next, we extend the utility of this approach in predicting the protein-protein association pathways based on available structures of individual subunits. We analyze the previously published MD simulations of the association of *E. coli* molybdopterin synthase subunits MoaD and MoaE^42^. Our results suggest that SAXS data must be combined with distance restraints, which are inferred from intermolecular ECs to better distinguish degenerate MoaD-MoaE structures displaying similar SAXS profiles. Using kinetic Monte Carlo sampling on the MSM built using the simulation datasets, we demonstrate that the utility of SAXS data in adaptive sampling can still reduce the computation time to reach the target structure. Furthermore, the sampling efficiency is further enhanced by utilizing both SAXS and distance restraints. Finally, we demonstrate the application of this approach in actual MD simulations to study the association of homodimer of plant hormone receptor PYR1^43^. As in MoaD-MoaE association, by combining SAXS information and distance restraints, we discover a structure that aligns well with the crystal structure (*C*_*α*_ RMSD: 3.18 Å) with a limited sampling time. Our study demonstrates that SAXS-guided adaptive sampling is an efficient approach to predict not only near-native structure ensemble but also transition pathways of conformational changes of proteins from simulation.

## Methods

### SAXS-guided adaptive sampling

The pipeline of adaptive sampling consists of iteratively running short parallel simulations, clustering the trajectories based on some structural features, and seeding new simulations from certain clusters according to some selection criterion^36^. The key of SAXS-guided adaptive sampling is to incorporate the SAXS information in the selections of seeding structures for iterative sampling. This is achieved by converting the SAXS profile into a SAXS discrepancy scoring function, which measures the degree of similarity between the target experimental or theoretical SAXS profile and the SAXS profile calculated from the structural models of each cluster. By selecting the clusters which are closer to the target, we bias the sampling direction while leaving the energy function unchanged. By iteratively running short parallel simulations, we drive the system of interest towards the target structure while still maintaining accurate thermodynamics and kinetics in the sampling process. The SAXS discrepancy function used in this study is the commonly used reduced *χ*^2^ function (equation 1):

1 $${\chi }^{2}=\frac{1}{N-1}\sum _{i=1}^{N}{(\frac{\mu {I}_{state}({q}_{i})-{I}_{target}({q}_{i})}{{\sigma }_{target}({q}_{i})})}^{2}$$

where *q*_*i*_ is the momentum transfer (*q* = 4*π*sinθ/λ, 2θ is the scattering angle and *λ* is the x-ray wave length), *I*_*target*_(*q*_*i*_) and *I*_*state*_(*q*_*i*_) are the scattering intensities of target SAXS profile and each cluster state at *q*_*i*_, *σ*_*target*_(*q*_*i*_) is the error of target scattering intensity at *q*_*i*_, *N* is the total number of data points in the SAXS scattering curves, *μ* is a scaling factor.

### Markov state model

Markov state model is a kinetic network model built from MD simulation datasets to describe the protein conformation space with discrete states and their transition probabilities^39,40^. The discrete states are generated from the clustering of all protein conformations based on some relevant structural metrics. The transition probabilities between these states are estimated by a maximum likelihood analysis of the interstate transition counts from the raw trajectories^44^. For an *N* state model with a lag time *τ*, the behavior of any initial probability distribution ***p***(*t*) over time can be given by (equation 2):

2 $${\boldsymbol{p}}(t+k\tau )={\boldsymbol{p}}(t){\boldsymbol{T}}{(\tau )}^{k}$$

where ***p***(*t*), ***p***(*t* + *kτ*) are *N* dimensional vectors of the state probabilities, ***T***(*τ*) is the transition probability matrix with each component *T*_*ij*_ as the transition probability between state *i, j* at a period of *τ*. The eigenvectors of ***T***(*τ*) in descending order by eigenvalues represent approximations of the underlying continuous-space propagator, where the first eigenvector is the equilibrium state probability vector ***w***^45^. The free energy of each MSM state *i* (*G*_*i*_) can be estimated by *G*_*i*_ = −*RTln*(*w*_*i*_), where *R* and *T* are the gas constant and temperature. Using MSMs, long timescale behavior of protein dynamics can be accurately predicted. All the MSMs in this study were constructed using MSMBuilder 3.4 package^46^.

### Kinetic Monte Carlo (MC) sampling on MSM

Kinetic MC simulation is a probabilistic method based on MSM transition probability matrix to generate arbitrary trajectory to reveal long term state-to-state protein dynamics^41^. If the initial state is chosen as state *i*, the probability of a transition from state *i* to state *j* over a period of *τ* is *T*_*ij*_. This is implemented by generating a pseudorandom number between 0 and 1, and taking a cumulative sum of *T*_*ij*_ over *j* (${S}_{n}={\sum }_{j}{T}_{ij}$). If the random number lies between S_*n*_ and S_*n*+1_, then the state *n* + 1 will be added to the trajectory. All the kinetic MC simulations in this study were conducted using MSMBuilder 3.4 package^46^.

### Computation of SAXS profiles

For the HP35, the protein G, the *α*3D domain and the MoaD-MoaE systems, all the SAXS profiles were calculated from the protein structure coordinates using the Crysol software^47^ provided in the ATSAS software package^48^, version 2.7.2. The SAXS scattering intensities were calculated between 0–0.5 Å^−1^ with 51 points in total. The number of points were chosen larger than the number of Shannon channels, as given by *N*_*s*_ = *q*_*max*_*D*_*max*_/*π*, where *q*_*max*_ is the maximum scattering vector and *D*_*max*_ is the maximum diameter of protein^49,50^. The number of harmonics was set to 40 and the order of Fibonacci grid was set to 18, and the default values of all other parameters were used. In the Crysol, the solvation shell of biomolecule is approximated by a border layer of certain effective thickness with a density differed from the bulk^47^. The contrast of hydration shell was set to 0.03 e/Å^3^ (default) and the solvent density was set to 0.334 e/Å^3^ (default).

For the PYR1 homodimer, all the SAXS profiles were calculated from the explicit solvent structural models using the WAXSiS algorithm^51,52^. In the WAXSiS, a spatial envelope that encloses all conformational states of the biomolecule with sufficient distance *d*, as well as the solvation layer, is defined to allow for the calculation of SAXS profiles from explicit solvent structural ensembles, while at the same time reducing statistical noise and computational cost. The explicit solvent representation effectively considers the structured water pattern within the solute solvation shell, allowing for accurate calculation of the scattering intensities at wide angles^51^. In this study, we calculated the SAXS scattering intensities at *q* between 0 to 1 Å^−1^ with 101 points (larger than the number of Shannon channels^49,50^ *N*_*s*_ ≈ 22). The envolope distance *d* was 7 Å, which has been shown to be enough to ensure bulk-like solvent density at envelope surface^51^. The density of the solvent was corrected to match the experimental value 0.334 e/Å^3^ using the density correction scheme implemented in WAXSiS. For each absolute value of the scattering angle *q*, 1000 homogeneous scattering vectors *q*_*j*_ (*j* = 1, …, 1000) were used for numerical computation of spherical average scattering intensity *I*(*q*). All calculations were performed using the WAXSiS implementation in modified version of the GROMACS simulation software, version 4.62^53^.

R_*g*_ and D_*max*_ estimated from the calculated or experimental SAXS profiles were determined using Guinier analysis as implemented in the ATSAS software package^48^. In addition to using the reduced *χ*^2^ function to assess the similarity between the target and predicted SAXS profiles, we also employed the correlation map method as implemented in the ATSAS software package^48^, which does not rely on the estimation of the errors of target SAXS profile. Gaussian random noises were added to the target scattering intensities *I*_*target*_(*q*_*i*_) to account for the errors while using the correlation map method. For example, Gaussian random noise at *q*_*i*_ was generated by a random number from a normal distribution with mean of 0 and standard deviation of *σ*_*target*_(*q*_*i*_). The randomly generated Gaussian noise was then added to *I*_*target*_(*q*_*i*_).

## Model systems

### Folding of single domain proteins

The total MD simulation times for the folding of HP35 double norleucine mutant, protein G, and *α*3D domain analyzed in this study were approximately 294, 1154, 707 *μ*s respectively^37,38^. The HP35 folding trajectories were clustered using *k*-centers algorithm based on the root mean square deviations (RMSDs) of all heavy atoms from the HP35 crystal structure (PDB ID: 2F4K^54^), same as in the previous literature^37^. An MSM with 500 states and a lag time *τ* of 30 ns was constructed. The protein G and *α*3D domain folding trajectories were clustered using *k*-means algorithm based on the 100 slowest-relaxing degrees of freedom from linear combinations of all *ϕ*, *ψ* and *χ*_1_ dihedral angles using the time-lagged independent component analysis (tICA)^55^. MSMs with 500 states and lag times of 50 ns were constructed. The lag times were chosen based on the convergence of implied timescales (Supplementary Fig. S1a–c). For each state of the MSMs, 100 structures were randomly extracted from the simulation datasets to calculate the SAXS profile of the state.

In order to calculate the SAXS profiles of native states of HP35, protein G and *α*3D, 50 ns explicit-solvent MD simulations on the experimentally determined structures of HP35, protein G and *α*3D (PDB IDs: 2F4K^54^, 1MI0^56^, 2A3D^57^) were performed in Amber14 using the Amber ff14SB force field^58^. The structures were solvated with TIP3P water and Na^+^/Cl^−^ were added to the system with the salt concentration of 0.15 M using AmberTools15. Subsequently, 10000 steps of energy minimization and 2 ns equilibration were performed for each system. Simulations were performed with a 2 fs time step and maintained at 300 K, 1 atm using Berendsen thermo-barostat^59^. The SHAKE algorithm^60^ was applied to constrain the length of covalent bonds involving hydrogen atoms. The Particle-mesh Ewald method^61^ was used to treat the electrostatic interactions with a 10 Å cutoff distance. 100 snapshots from the 50 ns MD simulations were extracted to calculate the SAXS profiles, and the non-weighted average of the 100 SAXS profiles was calculated to serve as the target SAXS profiles for adaptive sampling. More specifically, at each scattering vector *q*_*i*_, the average of scattering intensities (*I*(*q*_*i*_)) and the standard deviation of *I*(*q*_*i*_) were determined as *I*_*target*_(*q*_*i*_) and *σ*_*target*_(*q*_*i*_), respectively. For all states, the SAXS discrepancy values were calculated using the reduced *χ*^2^ function.

Three different sampling strategies: (1) traditional long simulation, (2) random adaptive sampling and (3) SAXS-guided adaptive sampling were employed in kinetic MC sampling to compare their sampling efficiency for all three proteins. The initial state was the expanded unfolded state with the largest RMSD value from target structure. The total sampling times required to reach the predicted folded state from the initial state were calculated for these three sampling schemes. In traditional long simulations, varying number of parallel simulations (10 to 1000) starting from the initial state were run for varying amount of time (1*τ* to 15*τ*), and 1500 sets of synthetic simulations were run in total. In random adaptive sampling, 10 parallel trajectories were launched from the initial state and run for varying amount of time (1*τ* to 15*τ*). Then 10 new states were randomly chosen from the resulting trajectories as the seeds for next round of sampling. This process iterated for varying number of adaptive rounds (1 to 100) and again, 1500 sets of synthetic simulations were run in total. Lastly, SAXS-guided adaptive sampling was following exactly the same procedures as in random adaptive sampling except 10 seeds in each round were chosen from the states that give the lowest SAXS discrepancy values. The sampling times required to reach the predicted folded state were calculated to quantify the sampling efficiency using different protocols.

Finally, for HP35 and protein G, we also tested the sampling efficiency using SAXS-based hybrid information-guided sampling approach combining both SAXS and distance restraints inferred from intramolecular evolutionarily couplings (ECs). The ECs were extracted using a pseudolikelihood (PLM) method^62^ on EVCouplings web server^63^ (http://evfold.org) with the default settings. For each MSM state, the distances of top ranked evolutionarily coupled residue pairs (with EC score > 0.3, 10 ECs for HP35, and 7 ECs for protein G, Supplementary Fig. S2) were calculated. The average residue pair distances were used for adaptive sampling. Under SAXS-EC-guided adaptive sampling protocol, we iteratively chose 5 states with the minimal SAXS discrepancy scores and 5 states with the minimal EC residue pair distance for adaptive sampling. For comparison, we also tested the efficiency of EC-guided adaptive sampling^42,64^, where in each round, 10 states with the minimal EC residue pair distances were picked for adaptive sampling.

### MoaD-MoaE association

55 *μ*s of previously published implicit solvent MD simulations on MoaD and MoaE association were used in this study^42^. The trajectories were clustered into 500 states using *k*-means algorithm (see Supplementary Information for details). An MSM was constructed with a lag time *τ* of 40 ns chosen based on convergence of the implied timescales (Supplementary Fig. S1d). The SAXS profile of the native MoaD-MoaE complex was calculated from 100 snapshots of 50 ns implicit solvent MD simulation on the MoaD-MoaE complex crystal structure (PDB ID: 1FM0^65^), which was also taken from the previous study^42^. At each scattering verctor *q*_*i*_, the average of scattering intensities (*I*(*q*_*i*_)) and the standard deviation of *I*(*q*_*i*_) were determined as *I*_*target*_(*q*_*i*_) and *σ*_*target*_(*q*_*i*_), respectively. 100 structures from each state were randomly extracted to calculate the SAXS profiles of each state. The SAXS discrepancy values between the SAXS profiles of each state and the target were calculated using the reduced *χ*^2^ function. The average SAXS discrepancy value of each state was calculated from the lowest 50 discrepancy values, in order to reduce the statistical errors due to clustering.

The distances of five evolutionarily coupled MoaD-MoaE residue pairs (E12-R127, R11-E53, A54-M58, Q57-K61, T58-K61) with the highest EVcomplex scores determined in the previous study^66^ were also calculated from the 50 structures with the lowest SAXS discrepancy scores from each state. Different kinetic MC sampling algorithms were carried out following the same protocol as in the folding systems. The initial state was the state with the largest average residue pair distance and the predicted dimeric state was chosen as the final state. The sampling times required to reach the predicted dimeric state were calculated to quantify the sampling efficiency using different protocols.

### Homodimeric PYR1 association

SAXS-guided MD simulations were performed to predict the dimeric PYR1 structure. We performed 10 ns explicit solvent MD simulations on the PYR1 crystal structure (PDB ID: 3K3K^43^) with and without position restraints on protein heavy atoms. The resulting trajectories (each with 1000 frames) were given as the input of the WAXSiS to calculate their theoretical SAXS profiles. The errors of the calculated SAXS profiles were determined by the WAXSiS automatically. The experimental SAXS data adapted from Nishimura *et al*.^43^ (Bioisis ID: BID_1PYR1P, http://www.bioisis.net/experiments/44) were fitted to the calculated SAXS profiles by minimizing the following *χ*^2^ function (on logarithmic scale, equation 3) as implemented in WAXSiS^51,52^:

3 $${\chi }^{2}=\frac{1}{N}\sum _{i=1}^{N}{[log{I}_{cal}({q}_{i})-log(f{I}_{exp}({q}_{i})+c)]}^{2}$$

where *N* is the total number of data points, *q*_*i*_ is the momentum transfer, *I*_*cal*_(*q*_*i*_) and *I*_*exp*_(*q*_*i*_) are the calculated and experimental scattering intensity at *q*_*i*_, *f* and *c* are the fitting parameters. We find that the experimental SAXS data fit better to the SAXS profile calculated from free MD simulations with a *χ*^2^ of 0.006 (as compared to 0.010 for the SAXS profile calculated from constraint MD simulations, Supplementary Fig. S3). The SAXS profile calculated from free MD simulations was used for the final structure identification.

Initially, two monomers of the crystal structure were separated with their center of mass distance approximately at 50 Å in VMD^67^. The structures were solvated with TIP3P water and Na^+^/Cl^−^ were added to the system with the salt concentration of 0.15 M using AmberTools15. The simulations were performed in Amber14 using the Amber ff14SB force field^58^. The simulation protocol and parameters were the same as described in the MD simulations of experimentally determined structures of single domain proteins. A single simulation was started from the equilibrated structure and run for 100 ns and the resulting trajectory was clustered into 100 states using a similar clustering scheme as in the MoaD-MoaE system. The SAXS profiles of all states were calculated. 25 clusters with the lowest SAXS discrepancies (calculated using *χ*^2^ function on logarithmic scale) from the target SAXS profile were chosen as the seeding structures for the second round of sampling. In the second round, each trajectory was run for 100 ns. The trajectories were clustered into 200 states, and again 25 clusters with the lowest SAXS discrepancy scores were chosen for the third round of sampling. In the third round, each trajectory was run for 60 ns, yielding total simulation time of around 4 *μ*s. Trajectories in the last two rounds were clustered into 200 states for final analysis. The SAXS profiles of these 200 states were calculated and the SAXS discrepancy scores were computed using the reduced *χ*^2^ function for consistency. In order to further improve the accuracy of the structural model obtained from the three rounds of adaptive sampling, 5 parallel simulations were run for 20 ns each starting from the closest near-dimeric state among the 200 states.

## Results

### SAXS along with thermodynamic information is sufficient to distinguish the native folds of small proteins

As an illustration of utilizing SAXS information to predict the native state structure of proteins, we first studied the folding of HP35 double norleucine mutant, protein G and *α*3D domain. The three systems with varying number of residues (35, 56, 73) were chosen to explore how the prediction accuracy changes as protein size increases due to the inherent ambiguity of SAXS data. MSMs were constructed for each system from the extensive amount of folding trajectories. The SAXS profiles calculated from short MD simulations on the experimentally determined structures or the structure of the closest homolog in the PDB (PDB IDs: 2F4K^54^, 1MI0^56^, 2A3D^57^) were used to obtain the target SAXS profiles for structure prediction. The SAXS discrepancy scores (reduced *χ*^2^) between the SAXS profile of each state and the target SAXS profile were computed. To compare the SAXS discrepancy scores of all states, we can predict the near-folded state and further test whether SAXS is sufficient to make good predictions by aligning the predicted structure to the experimentally determined structure.

**Figure 1:**
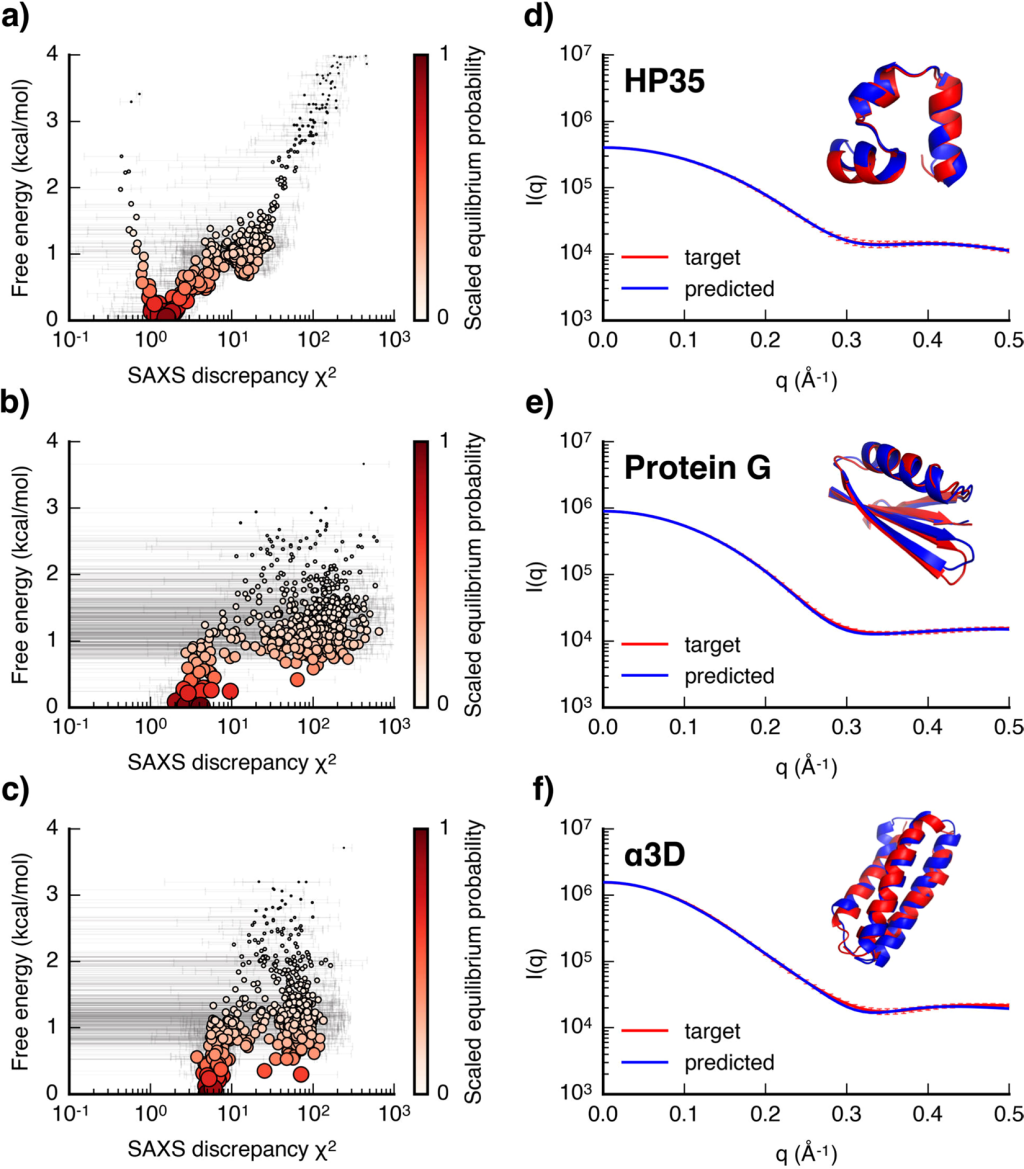
Predicting native folds of single domain proteins. The plots of individual MSM state free energy with respect to their average SAXS discrepancy values (*χ*^2^) for the (**a**) HP35, (**b**) protein G, and (**c**) *α*3D. Each dot represents a MSM state. The dot size and its color darkness are scaled by the equilibrium probability of that state estimated from the MSM. The errorbars for the SAXS discrepancy values of all states are shown in grey line. SAXS profiles of the target (red) and the predicted (blue) states, and overlays of the crystal structure (red) and the simulation predicted structure (blue) for (**d**) HP35, (**e**) protein G, and (**f**) *α*3D. The errorbars for the target SAXS profiles are also shown in the figure.

This is shown by plotting the free energies of all MSM states with respect to their SAXS discrepancy scores, as shown in Fig. 1a–c. Without any *a priori* knowledge, the free energy information estimated from the MSMs can serve as additional metric to identify the near-native or intermediate states of proteins from simulation. Combining both free energy and SAXS discrepancy information, we seek to make prediction of the native folds of proteins. For these three folding systems, generally, high free energy states tend to have much higher SAXS discrepancy values, while low free energy states corresponding to more stable near-native or intermediate structures tend to have much lower SAXS discrepancy values (Fig. 1a–c). The RMSD plots in Supplementary Fig. S4 also suggest that the states with high SAXS discrepancy scores have large C_*α*_ RMSDs from their crystal structures, and *vice versa*. Though in HP35 a few states with minimal SAXS discrepancy scores have high free energy (Fig. 1a), these structures are actually close to the native structures (Supplementary Fig. S4). However, as protein size increases, there are more low free energy states (<1 kcal/mol) with comparably low discrepancy values in *α*3D as compared to the states in smaller HP35 and protein G, which will be hard to distinguish using SAXS information alone. This is consistent with the underdetermined nature of SAXS pattern. All together, this suggests that for the folding of small single domain proteins, thermodynamic information estimated from the MSMs is valuable for dealing with the ambiguity of SAXS data.

Based on both free energy and SAXS discrepancy information, the states with the lowest free energy were predicted as the near folded states from the simulation datasets. The SAXS profiles of the target and the predicted folded states for the three proteins are shown in Fig. 1d–f. The predicted post-MD model SAXS profiles of HP35, protein G and *α*3D fit to their target SAXS profiles with reduce *χ*^2^ values of 0.045, 0.775 and 0.975. The residuals plots suggest that the discrepancies between the predicted and the target SAXS profiles are comparable to the errors on the target SAXS profile (Supplementary Fig. S5). The fittings were also assessed using the correlation map method^68^, which also suggest high similarities between the target and predicted SAXS profiles (Supplementary Fig. S6). Overlays of the native structures and the predicted folded structures of HP35, protein G and *α*3D give the C_*α*_ RMSDs of 0.7, 0.78 and 2.75 Å respectively. Radius of gyration (R_*g*_) and maximum diameter (D_*max*_) estimated from the target and predicted SAXS profiles using Guinier analysis are in good agreement (Table 1).

**Table 1:** Comparisons of the radius of gyration (R_*g*_) and the maximum diameter (D_*max*_) of the native and the predicted states estimated from the SAXS data.

Systems	R_*g*_ (Å)		D_*max*_ (Å)	
	native	predicted	native	predicted
HP35	10.96	10.90	35.61	33.29
Protein G	12.25 ± 0.01	12.27 ± 0.01	37.04	39.76
*α*3D	14.34 ± 0.01	14.61 ± 0.02	45.58	45.32
MoaD-MoaE	21.38	21.55 ± 0.03	72.67	76.07
PYR1	23.89 ± 0.08 (calc.)23.72 ± 0.6 (expr.)	23.92 ± 0.07	67.4 (calc.)68.46 (expr.)	67.57

An accurate estimate of free energy values from the MSMs usually requires sufficient amount of sampling. To further improve the accuracy of identifying native structures from simulation datasets, other types of structural information can be incorporated together with SAXS data. For example, we demonstrate that in combination with the distance restraints inferred from a few top ranked evolutionarily coupled residue pairs, the near-native states of HP35 and protein G can be identified (Supplementary Fig. S2). This hybrid information could tackle with the challenge of SAXS inherent ambiguity and possible thermodynamic inaccuracy due to insufficient sampling.

### Enhanced efficiency in sampling protein folding

**Figure 2:**
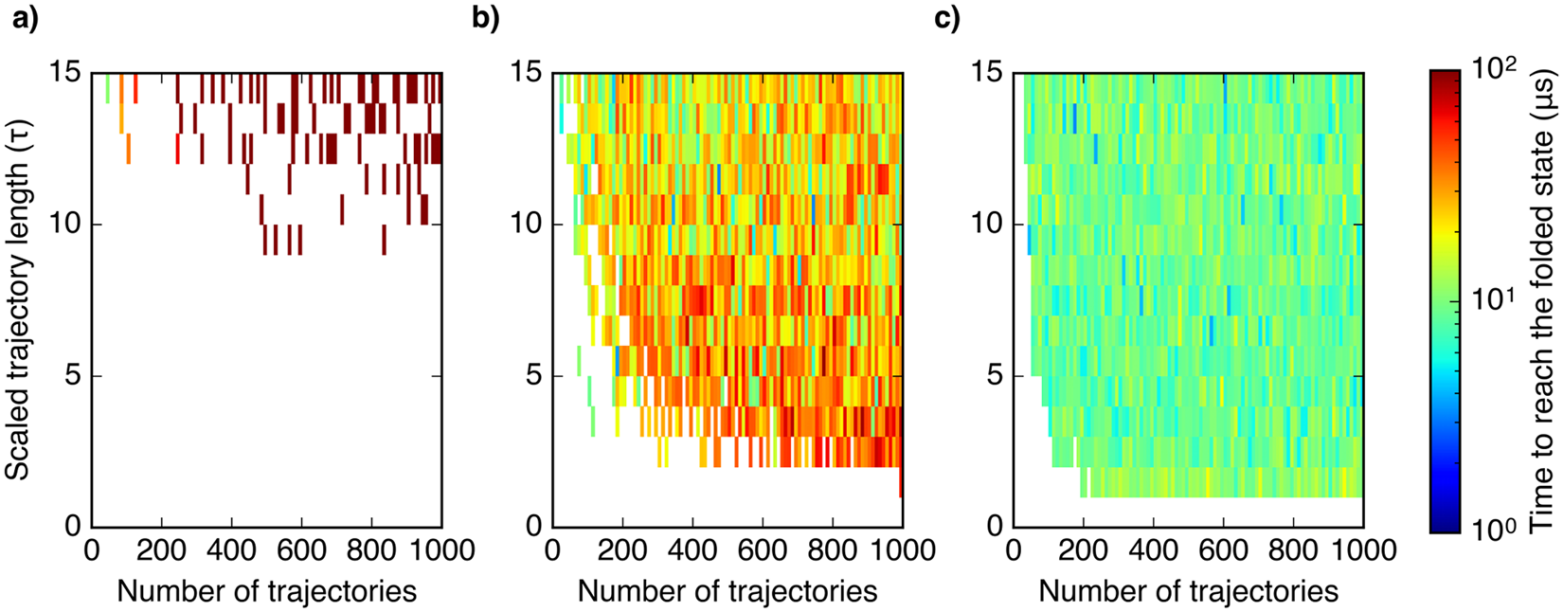
Enhanced efficiency in sampling the folding of HP35. Total simulation time required to reach the folded state from an arbitrary unfolded state for sets of samplings using (**a**) traditional long simulation, (**b**) random adaptive sampling, and (**c**) SAXS-guided adaptive sampling. Scaled trajectory length is the length of each individual trajectory in each specific sampling scheme by the lag time *τ* of the MSM. Number of trajectories is the total number of trajectories run for each sampling scheme, given by the product of the number of parallel trajectories and the number of sampling rounds. The average total required sampling times using the 3 different protocols over 1500 sets of samplings (excluding the sets of sampling that do not reach the target native state) are 235.03 *μ*s, 27.61 *μ*s, 9.76 *μ*s.

To test the efficiency of utilizing the SAXS discrepancy information in sampling protein folding, we employed kinetic MC simulations of the folding of HP35, protein G and *α*3D on the MSMs using different sampling strategies. The total sampling times required for transition from an arbitrary expanded unfolded state to the predicted folded state were calculated to compare the overall sampling efficiency of different sampling strategies. Figure 2 shows the results for sampling the folding of HP35 using traditional long simulation, random and SAXS-guided adaptive samplings. It is clearly shown in Fig. 2a that traditional way of running long simulations takes the longest time to discover the folded state. Few simulation sets with individual trajectory length shorter than 10 *τ* (300 ns MD simulation) can reach the folded state even with 1000 trajectories running in parallel. Adaptive sampling can effectively reduce the simulation time to reach the folded state. With random adaptive sampling (Fig. 2b), namely randomly picking seeds for iterative sampling, an order of magnitude decrease of computational time to reach the folded state over traditional long simulation is observed. In addition, in the short individual trajectory length regions where traditional long simulation sets can never reach the folded state, random adaptive sampling can reach the folded state in tens of microseconds. Figure 2c shows SAXS-guided adaptive sampling can even further decrease the sampling time than random adaptive sampling. The total simulation time required for obtaining the native state is ∼10 *μ*s. Enhanced efficiency in SAXS-guided adaptive sampling is also observed in sampling protein G and *α*3D (Supplementary Figs. S7 and S8). Using another metric, the number of MSM states explored using random and SAXS-guided adaptive sampling, to compare their sampling efficiency, it is clearly shown that SAXS-guided sampling enhances sampling efficiency by reducing the sampling of ‘insignificant’ states (Supplementary Fig. S9), which have large deviations from the target as measured by SAXS-discrepancy scores. Overall, these results suggest that utilizing the SAXS information in adaptive sampling can effectively reduce computational time required to discover the folded structures of small proteins and the folding pathways in unbiased simulations. We further tested the efficiency of utilizing both SAXS and distance restraints inferred from ECs in adaptive sampling of protein folding of HP35 and protein G (Supplementary Figs. S7 and S10). As compared to SAXS-guided or EC-guided adaptive sampling, we further observe a slight enhancement of sampling efficiency using this hybrid approach (Supplementary Figs. S7 and S10).

### SAXS along with distance restraints predict the near-crystal structure of MoaD-MoaE complex

The association of *E. coli* molybdopterin synthase subunits, MoaD and MoaE, was used to explore the application of SAXS-guided adaptive sampling approach in predicting dimeric protein structures and association pathways. As in the folding systems, the SAXS profiles of all MSM states were calculated, and the SAXS profile calculated from short MD simulations of the crystal structure (PDB ID: 1FM0)^65^ was used as the target SAXS profile. We first tested whether SAXS in combination with thermodynamic information is sufficient to make good predictions of dimeric structure from MD simulation datasets by comparing the SAXS profiles of each state and the target. Supplementary Fig. S11 gives the plots of free energies of all states with respect to their SAXS discrepancy scores. Unlike the free energy plots in folding systems, there is not a clear correlation between the free energy of each state and its SAXS discrepancy value. Although several most populated states estimated by the MSM have relatively low SAXS discrepancy values, there are many less populated states that can give even lower SAXS discrepancy values, which might imply their higher structure similarity to the target structure. From this, we speculate that the most populated state might not be the actual near-dimer structure but a thermodynamically metastable state, which is possible considering the insufficient sampling of the protein-protein association ensembles.

**Figure 3:**
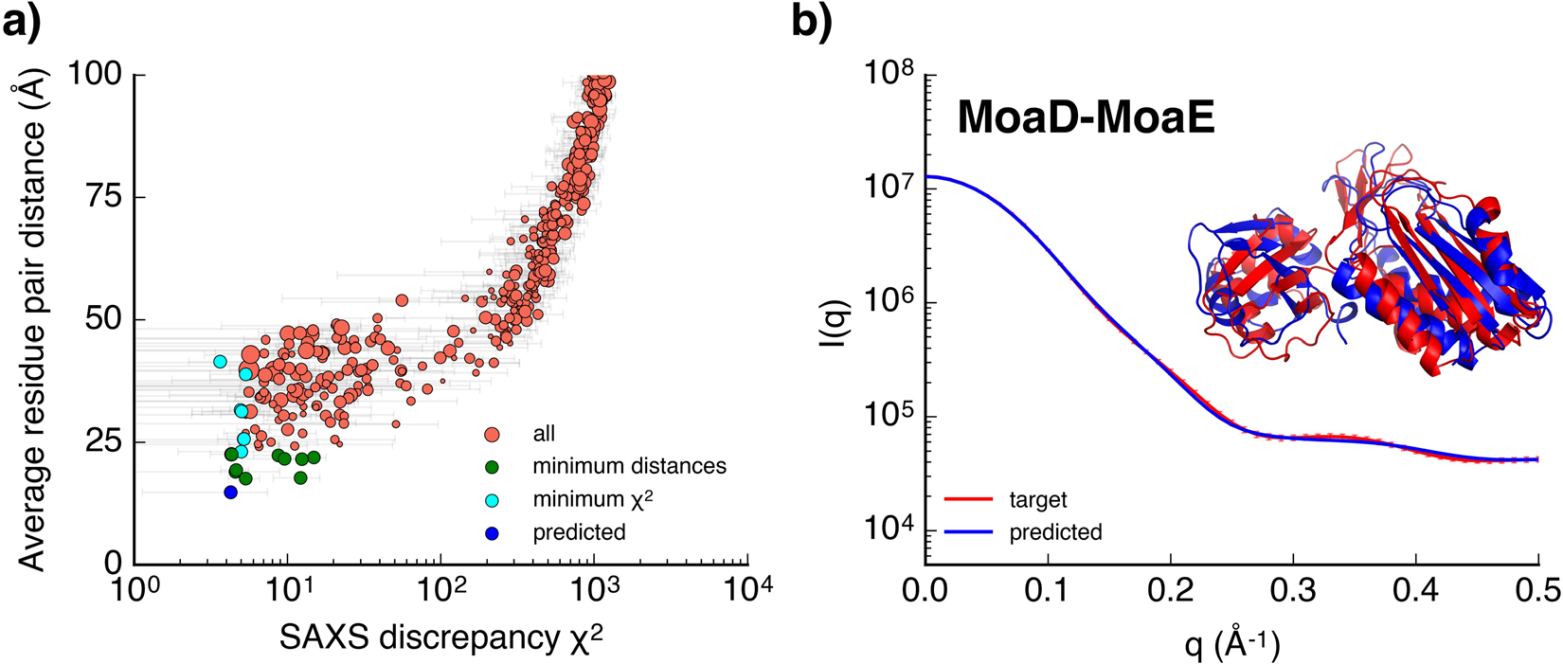
Predicting the structure of MoaD-MoaE complex. (**a**) The plots of the average *C*_*α*_ distance of five evolutionarily coupled residue pairs of each MSM state with respect to their average SAXS discrepancy scores (*χ*^2^). Each dot represents an MSM state. The dot size is scaled by the equilibrium probability of that state estimated from the MSM. The 10 states with minimal average residue pair distances and the 10 states with minimal *χ*^2^ are colored in green and cyan, respectively (with 3 overlapped states colored in green and the overlapped predicted state colored in blue). (**b**) SAXS profiles of the target (red) and the predicted (blue) states. The errorbars for the target SAXS profile are also shown in the figure. Overlay of the crystal structure (red) and the simulation predicted structure (blue) gives a *C*_*α*_ RMSD of 5.32 Å from the crystal structure.

This prompts us to look for additional information to further distinguish the states displaying similar SAXS profiles. We explored to combine the distance restraints inferred from intermolecular ECs with SAXS data to improve structure prediction accuracy. Intermolecular ECs can provide valuable insights into residue contacts at protein-protein interface^66^. Top 5 five ranked EC residue pairs were chosen, and the distances between these residue pairs for all states were calculated. We plotted the average residue pair distances of all states with respect to their SAXS discrepancy scores to characterize their structure differences (Fig. 3a). We observe that there is still an overall correlation between the average residue pair distance and the SAXS discrepancy scores, though states with approximately equal SAXS discrepancy scores can have large varying average residue pair distances. For example, the 10 states with the minimal SAXS discrepancy scores (cyan, Fig. 3a) have significant differences in interfacial residue pair distances (range between 15–40 Å). As shown in Supplementary Fig. S12, these states all display similar SAXS profiles as compared to the target SAXS data, however, the MoaD-MoaE complexes adopt completely different orientations. The inherent ambiguity of SAXS data is much more obvious for MoaD-MoaE complex as compared to smaller single domain proteins. Nevertheless, incorporating distance restraints information at the complex interface effectively distinguish the states that display equally consistent SAXS profiles as the target SAXS profile.

Integrating both SAXS discrepancy scores and distance restraints information, we predict the state with the lowest interfacial residue pair distance, which also has relatively low SAXS discrepancy score, as the near-dimer structure (blue, Fig. 3a). Figure 3b shows the predicted structure aligns well with the crystal structure (*C*_*α*_ RMSD: 5.32 Å) and the SAXS profile of the predicted structure also matches well with the target SAXS profile (reduced *χ*^2^ = 0.922, residuals plots shown in Supplementary Fig. S13). Fitting assessed using the correlation map method also suggests that the predicted SAXS profile adequately describes the target SAXS data (Supplementary Fig. S14). The relatively large RMSD could be due to the loop of MoaD that is inserted into MoaE to form the active site in the crystal structure^65^. This process has not been captured from the dataset^42^. The R_*g*_ and D_*max*_ values estimated from the target and predicted SAXS profile are also in good agreement (Table 1).

Supplementary Fig. S15 shows the comparison of the SAXS profiles and the snapshots of the 10 states with the minimal average residue pair distances (green, Fig. 3a). As compared to the predicted state, the states with relatively higher SAXS discrepancy scores show larger RMSDs from the crystal structure. A combination of SAXS and interfacial residue contact information gives the best structure prediction. All together, these results demonstrate that a hybrid approach that combines SAXS with distance restraints information provides a good structure prediction of protein-protein complex.

### Enhanced efficiency in sampling protein-protein association

**Figure 4:**
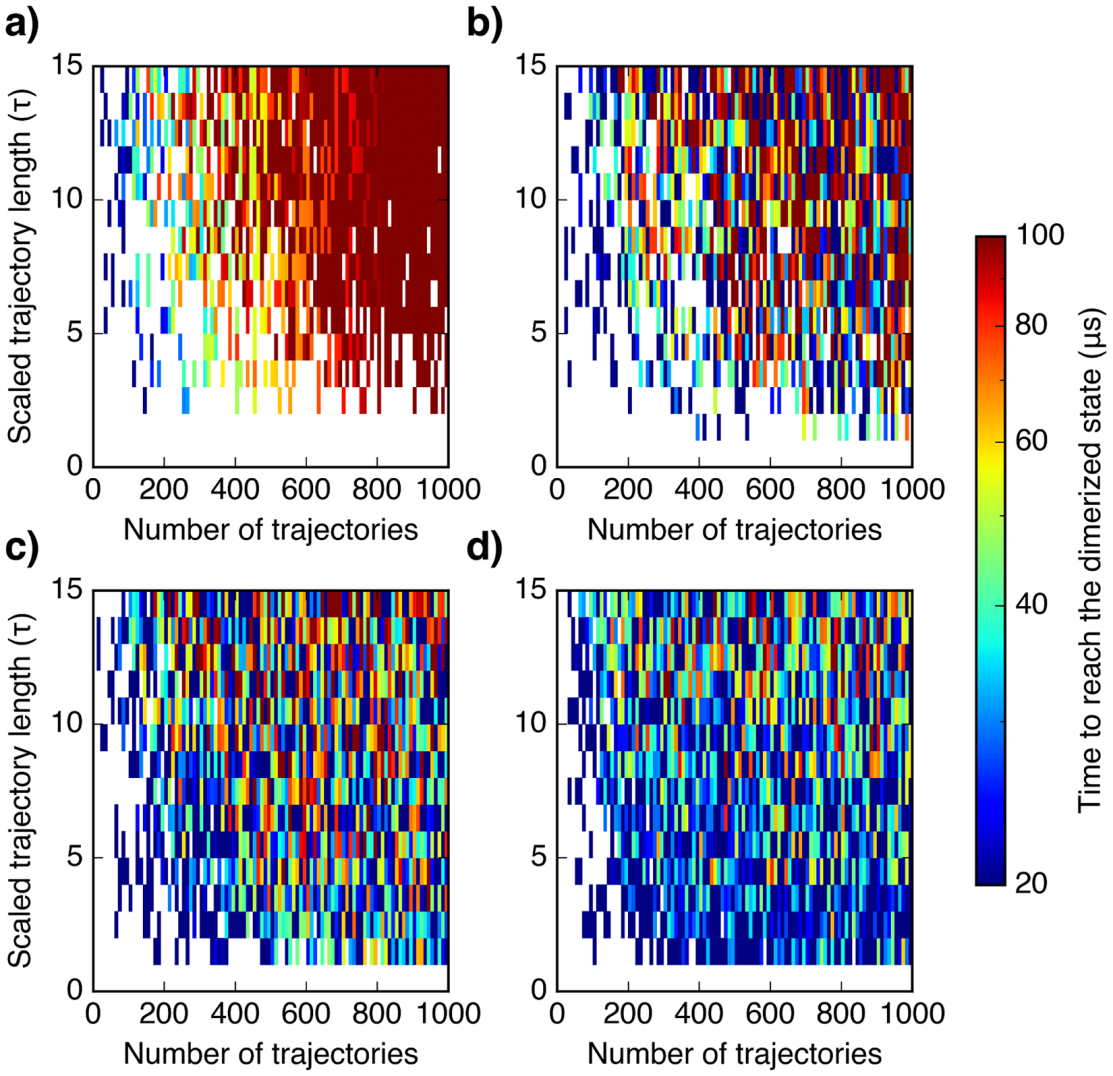
Enhanced efficiency in sampling the association of MoaD-MoaE. Total simulation time required to reach the predicted dimeric state from an arbitrary unassociated state for sets of samplings using (**a**) traditional long simulation, (**b**) random adaptive sampling, (**c**) SAXS-guided adaptive sampling and (**d**) SAXS-EC-guided adaptive sampling. Scaled trajectory length is the length of each individual trajectory in each specific sampling scheme by the lag time *τ* of the MSM. Number of trajectories is the total number of trajectories run for each sampling scheme, given by the product of the number of parallel trajectories and the number of sampling rounds. The average total required sampling times using the 4 different protocols over 1500 sets of samplings (excluding the sets of samplings that do not reach the target state) are 113.28 *μ*s, 61.16 *μ*s, 41.57 *μ*s, and 30.64 *μ*s.

In order to test the feasibility of utilizing SAXS to accelerate unbiased sampling of protein association pathways, we performed kinetic MC samplings on the MoaD-MoaE MSM using different protocols, including traditional long simulation, random adaptive sampling, SAXS-guided as well as SAXS-EC guided adaptive samplings. We calculated the total sampling time required to observe the transition from an arbitrarily chosen unassociated state to the predicted near-dimeric state, as shown in Fig. 4. As in the folding systems, adaptive sampling strategy effectively reduces the total sampling times as compared to long serial simulations. As compared to random adaptive sampling, SAXS-guided adaptive sampling also improves the sampling efficiency. In the 1500 sets of samplings, the average total sampling time to reach the target state is ∼60 *μ*s using random adaptive sampling, and the required sampling time decreases to ∼40 *μ*s using SAXS-guided sampling. In the case of hybrid approach using SAXS-EC-guided adaptive sampling, we observe a further improvement of the sampling efficiency, and the average required sampling time is ∼30 *μ*s. When compared with the performances of SAXS-guided or EC-guided adaptive sampling, SAXS-EC-guided adaptive sampling performs better as compared to both sampling strategies (Supplementary Fig. S16). All together, these results suggest that SAXS-guided sampling approach enhances time efficiency in sampling protein-protein association, and by combining both SAXS and distance restraints, the sampling efficiency is further enhanced.

### SAXS-guided adaptive sampling provides a near-crystal structure PYR1 complex

The last example is to test the efficiency of using the SAXS-guided adaptive sampling in actual MD simulations to predict the complex structure of PYR1. The target SAXS data was computed from short explicit-solvent MD simulations on the PYR1 crystal structure (PDB ID: 3K3K)^43^. After each round of sampling, all protein conformations were clustered and the SAXS discrepancy scores of all states were calculated and used in the adaptive sampling. By the third round of sampling, the structure with *C*_*α*_ RMSD from the crystal structure of 5.14 Å was achieved, with total sampling time of 4 *μ*s. The trajectories in last two rounds were clustered into 200 states, and the SAXS profiles of these 200 states were computed and the SAXS discrepancy scores between the target and each state were calculated using the reduced *χ*^2^ function. Two pairs of residues from each monomer (K63-D155, L166-L166) at the interface of the crystal structure were chosen to calculate the distances to characterize the structural similarity to the crystal structure.

**Figure 5:**
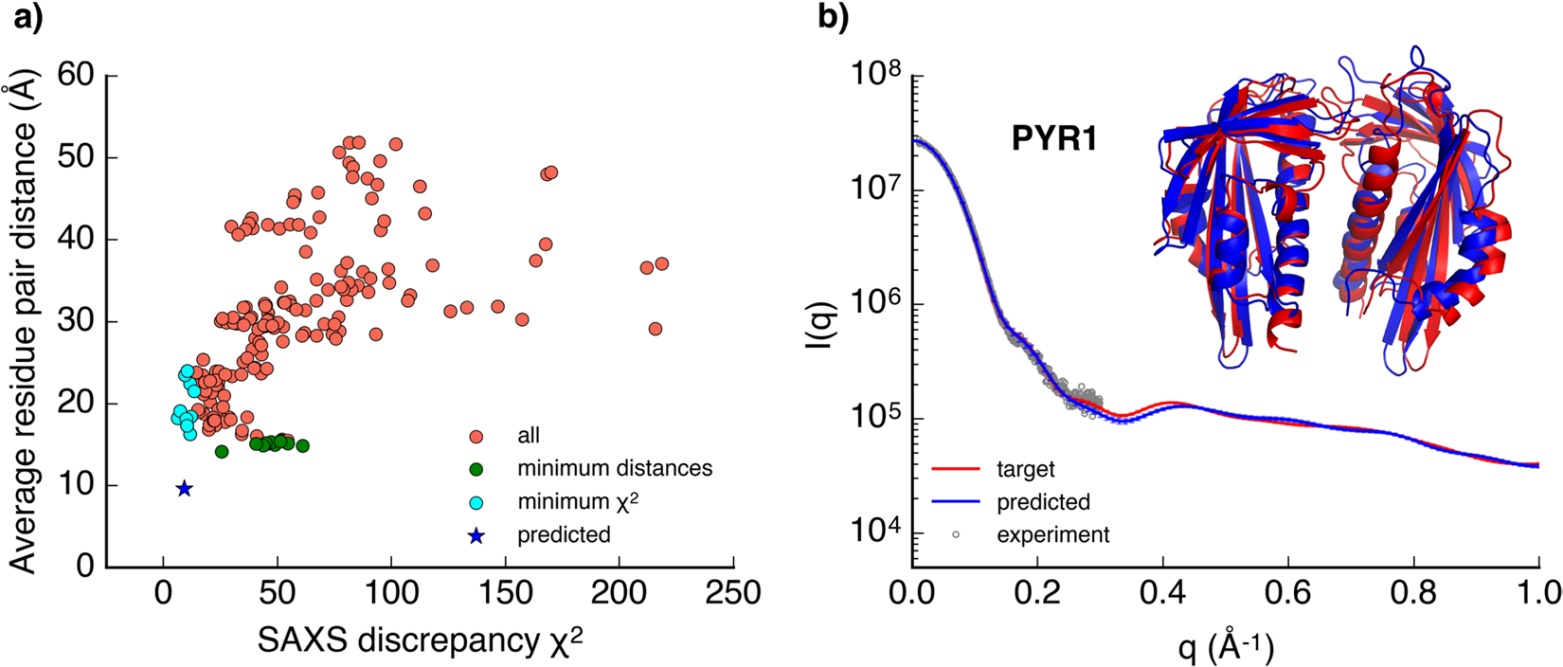
Predicting the PYR1 structure. (**a**) The plot of the average *C*_*α*_ distance of two residue pairs of individual state with respect to the SAXS discrepancy value *χ*^2^. Each circle represents a single state from the clustering. The 10 states with the minimal average residue pair distances are colored in green, and the 10 states with the minimal *χ*^2^ are colored in cyan, respectively. The blue star denotes the refined predicted PYR1 structure obtained after 20 ns MD simulation starting from the state with the minimal residue pair distance. (**b**) SAXS profiles of the target (red) and the predicted (blue) states are shown with the errorbars. Overlay of the crystal structure (red) and the best simulation predicted structure (blue) gives a *C*_*α*_ RMSD of 3.18 Å. The fitted SAXS experimental data are marked in grey circles.

Figure 5a gives the plots of average residue pair distances of all states with respect to their SAXS discrepancy values. Similar to the association of MoaD and MoaE, even at low SAXS discrepancy region, there are multiple states that have approximately equal SAXS discrepancy values but varying residue pair distance; while the structures with approximately equal average residue pair distances can have varying SAXS discrepancy values. Supplementary Fig. S17 shows the SAXS profiles and the snapshots of the 10 states with the minimal SAXS discrepancy scores. Despite high similarities between the SAXS profiles of these states as compared to the target SAXS profile, the two monomers adopt various types of orientations and have varying degrees of deviations from the crystal structure. These results again highlight the inherent ambiguity of SAXS patterns. Supplementary Fig. S18 shows the SAXS profiles and the snapshots of the 10 states with the minimal interfacial residue pair distances. From the three rounds of adaptive samplings, the state with the lowest interfacial residue pair distance and a relatively small SAXS discrepancy score (reduced *χ*^2^ = 25.561) gives the closest structural model to the PYR1 crystal structure (C_*α*_ RMSD: 5.14 Å, Supplementary Fig. S18b).

To further improve the quality of the PYR1 structural model, we performed additional MD simulations to refine the structural model obtained from the three rounds of adaptive sampling. Briefly, 5 parallel simulations were launched from the state with the minimal residue pair distance (among the 200 states) and run for 20 ns each. The last 10 ns simulation data from each trajectory were used to calculate the SAXS profiles. The conformation from the trajectory with the lowest reduced *χ*^2^ was determined as the predicted PYR1 structural model (Fig. 5a, blue star). The reduced *χ*^2^ between the predicted and the target SAXS profiles decreases to 9.111 and the C_*α*_ RMSD of the predicted structural model from the crystal structure decreases to 3.18 Å (Fig. 5b). The target SAXS profile, and the SAXS profile of the predicted structure are shown in Fig. 5b. The target SAXS profile matches well with the previously published SAXS experimental data^43^ within q < 0.3 Å^−1^ region. The R_*g*_ and D_*max*_ values estimated from the experimental SAXS data and the target and predicted SAXS profiles are compared in Table 1. The residuals plots suggest that the major discrepancies between the predicted and the target SAXS profiles are from the errors of the predicted SAXS profiles (Supplementary Fig. S19). We believe more sampling from the predicted structural model will further improve the structural prediction accuracy and the corresponding SAXS profile will have even better match with the target SAXS profile. Overall, these results demonstrate that SAXS-guided adaptive sampling is an efficient sampling approach to predict protein complex structures from unbiased all atom MD simulations.

## Discussion

Long timescale unbiased MD simulations can be a complementary method to fully interpret the limited structural information contained in SAXS data, and predict accurate protein structures, ensembles and dynamics. In this study, we have demonstrated the utility of SAXS and hybrid information in adaptive sampling process to enhance time efficiency in unbiased sampling of protein folding and protein-protein association pathways. By analyzing the extensive protein folding and protein-protein association simulation datasets, we demonstrate the use of SAXS data along with thermodynamics or distance restraints information in improving the accuracy of structure prediction from MD simulations. For the folding of small proteins, we show that SAXS data in combination with free energy information estimated from the MSMs is sufficient to distinguish the native states from simulation datasets (Fig. 1). Distance restraints which can be inferred from intramolecular EC can be combined with SAXS data to further distinguish the internal structure differences of conformational states that display similar SAXS profiles. For the association of MoaD-MoaE (Fig. 3) and homodimer PYR1 (Fig. 5), integrating SAXS data and interfacial distance restraints, good predictions of near-native complex structures can be obtained using this approach. For practical applications, which additional external information may be required for further structure differentiations can also be obtained from these structural models predicted from the simulation. Based on this prior knowledge, relevant computations or experiments could be performed to provide the information^69–71^.

From kinetic MC sampling, we have shown that the computational times in sampling either protein folding (Fig. 2, Supplementary Figs. S7, S8) or protein-protein association (Fig. 4) are significantly reduced by incorporating SAXS and SAXS-based hybrid information as reaction coordinates in adaptive sampling. Furthermore, by combining both SAXS and distance restraints in adaptive sampling, the sampling efficiency is better than the sampling guided by either type of structural information alone. We expect that these hybrid approaches will be useful for the study of larger proteins, as the inherent ambiguity of SAXS data would be more significant. It should be noted that these approaches provide not only the final predictions of native states of proteins and complexes, but also structure ensembles and dynamics. During the sampling process, our protocol bias sampling directions towards the target to prevent exploring ‘irrelevant’ states as defined according to the adaptive seed selection criteria. After the target structure is discovered, one can do more sampling along the initial sampled pathways to collect accurate structural ensembles and dynamics. For heterogeneous ensembles, the obtained ensembles could be reweighted against experimental data to be further refined.

Our approach has some similarities to a previously proposed experiment-guided sampling technique, PaCS-Fit^72^. PaCS-Fit also involves iteratively running short parallel simulations and picking conformations that are ‘closer’ to experimental data for further sampling except the clustering step as implemented in our MSM-based adaptive sampling strategy. The clustering step is essential for two reasons. First, due to SAXS underdetermined nature, sturctures with similar molecular envelopes may display similar SAXS profiles but can have essential structure differences at atomic resolution. This is already demonstrated in our MoaD-MoaE and PYR1 systems. Without clustering, in each cycle PaCS-Fit will likely only select redundant conformations that are structurally similar to continue sampling, and therefore bias simulations to an ensemble consistent with target experimental data but structurally distant from target structure. Instead, by iteratively running simulations in parallel from multiple cluster states, we can likely achieve the structure ensemble reasonably ‘close’ to the true ensemble. Second, the clustering can effectively reduce the amount of SAXS calculations required for long timescale simulations. Peng *et al*.^72^ has demonstrated the success of application of PaCS-Fit in predicting small conformational changes of proteins. Our study has addressed some challenges as we extend the application of this approach in studying the protein folding and protein-protein association process.

Finally, using MSMs, the large amount of unbiased simulation data collected using adaptive sampling approaches could be merged to construct discrete protein dynamic models. Using transition path theory^73,74^, protein dynamics such as protein folding and protein-protein pathways can be fully mapped out from the MSMs. All together, SAXS-based adaptive sampling along with MSMs potentially allows us to predict not only the protein functional conformations but also the pathways of conformational changes with a reasonable computational cost. This method can be useful in determining the unknown structures of proteins and complexes. At the same time, the constructed models from simulation can be updated when more accurate or orthogonal experimental information is available^69,70^. Other experimental information can be incorporated in a similar manner to collectively enhance the sampling and improve the accuracy of prediction from the simulation. These methods provide a way to build a dynamic model of protein function consistent with the available experimental and computational data.

## Electronic supplementary material

Supplementary Information
