# Supplementary material for: SAXS-guided Enhanced Unbiased Sampling for Structure Determination of Proteins and Complexes
Source: Sci Rep. 2018 Dec 10;8:17748. doi: 10.1038/s41598-018-36090-z (PMC6288155; doi:10.1038/s41598-018-36090-z)
Supplement: Supplementary file 1 — Supplementary Information [file 41598_2018_36090_MOESM1_ESM.pdf]

Supplementary Information for: SAXS-guided Enhanced Unbiased Sampling for Structure Determination of Proteins and Complexes

Chuankai Zhao[1] **and Diwakar Shukla**[1,2,3,4*]

[1]Department of Chemical and Biomolecular Engineering, University of Illinois, Urbana, IL, 61801, United States
[2]Department of Plant Biology, University of Illinois, Urbana, IL, 61801, United States
[3]Center for Biophysics and Quantitative Biology, University of Illinois, Urbana, IL, 61801, United States
[4]National Center for Supercomputing Applications, University of Illinois, Urbana, IL, 61801, United States
[*]Correspondence and requests for materials should be addressed to D.S. (email: diwakar.shukla@shuklagroup.org)

ABSTRACT

Supplementary information includes the method details of clustering the MoaD-MoaE and PYR1 association systems and the supplementary figures.

Clustering of association of protein complexes

The association of protein complexes involves multiple degrees of freedom, including translation and orientation changes between two monomers, and individual structure flexibilities of single units. To featurize the trajectories, the following calculations were carried out:

1) At each frame of the trajectories, two sets of basis vectors $\{\boldsymbol{x}_1, \boldsymbol{y}_1, \boldsymbol{z}_1\}$ and $\{\boldsymbol{x}_2, \boldsymbol{y}_2, \boldsymbol{z}_2\}$ were defined from the C_α atom coordinates of three residues picked from each monomer unit. The three residues were chosen for the comparatively low root mean square fluctuations of C_α atoms. Supplementary Fig. S20 gives an example of clustering MoaD-MoaE system to explain this process.

2) To characterize the translation changes, projections of the center of mass distance vector $\boldsymbol{r}$ along $\{\boldsymbol{x}_1, \boldsymbol{y}_1, \boldsymbol{z}_1\}$ were calculated denoted by $\{dx, dy, dz\}$.

3) To characterize the orientation changes, the angles between $\{\boldsymbol{x}_1, \boldsymbol{x}_2\}$, $\{\boldsymbol{y}_1, \boldsymbol{y}_2\}$, $\{\boldsymbol{z}_1, \boldsymbol{z}_2\}$ were calculated denoted by $\{\alpha, \beta, \gamma\}$.

4) To characterize the internal structural flexibilities, the RMSD of each monomer from the crystal structure were calculated denoted by $\{\mathrm{RMSD}_1, \mathrm{RMSD}_2\}$.

Therefore, a vector with 8 dimensions denoted by $\{dx, dy, dz, \alpha, \beta, \gamma, \mathrm{RMSD}_1, \mathrm{RMSD}_2\}$ was defined to characterize the dimeric structure at each frame. Time-lagged independent components analysis (tICA) was performed to characterize the slowest processes in the system. Clusterings of the MoaD-MoaE and dimeric PYR1 associations were based on the slowest 8 and 4 tICs, respectively.

Supplementary figures

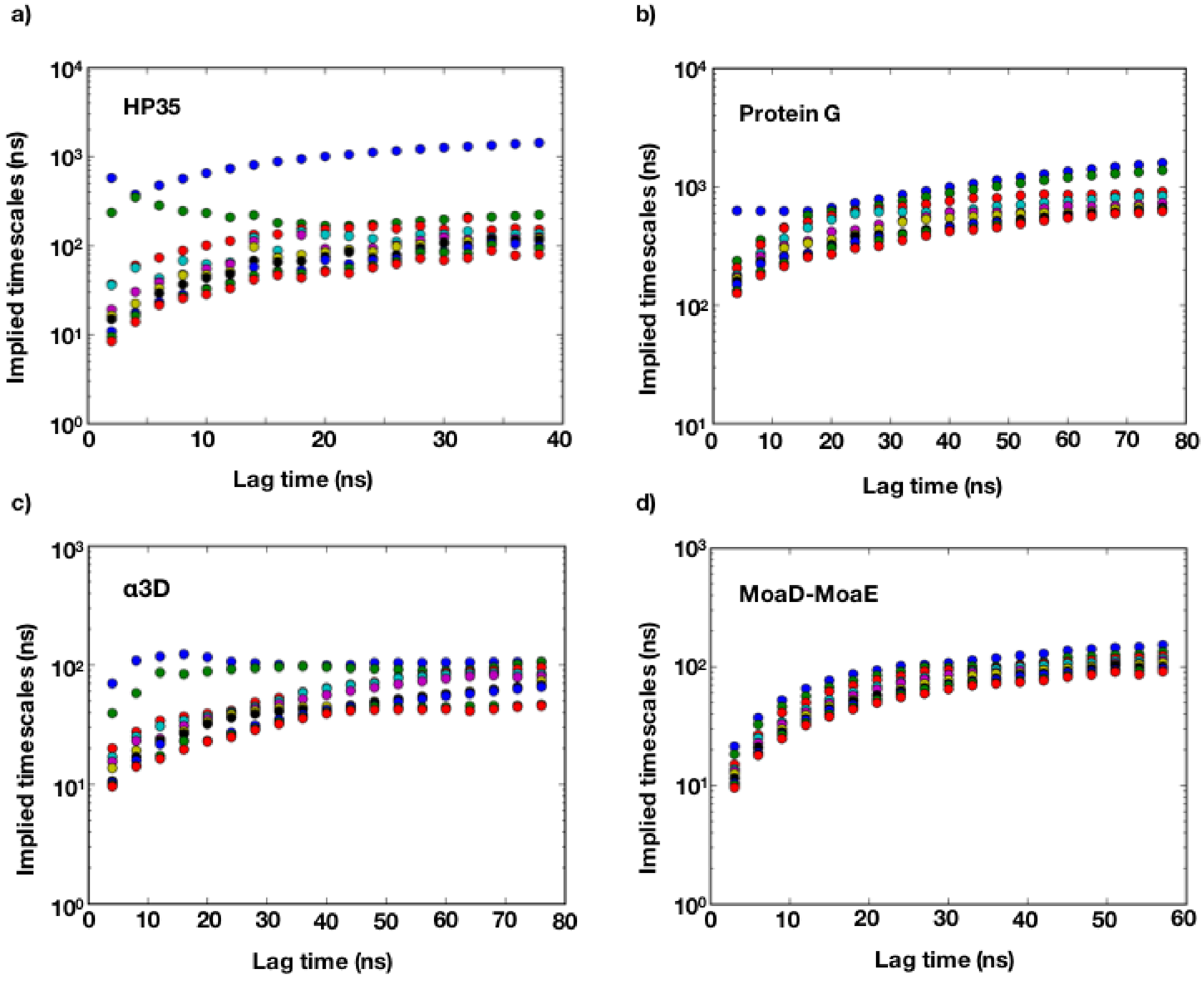

Figure S1. Implied timescales plots for the Markov state models. (a) HP35, (b) Protein G, (c) α3D, (d) MoaD-MoaE. The timescales converge to the true relaxation timescales with the increase of lag time τ, implying the Markovian behavior of the MSMs. The lag times were chosen as 30, 50, 50 and 40 ns, respectively.

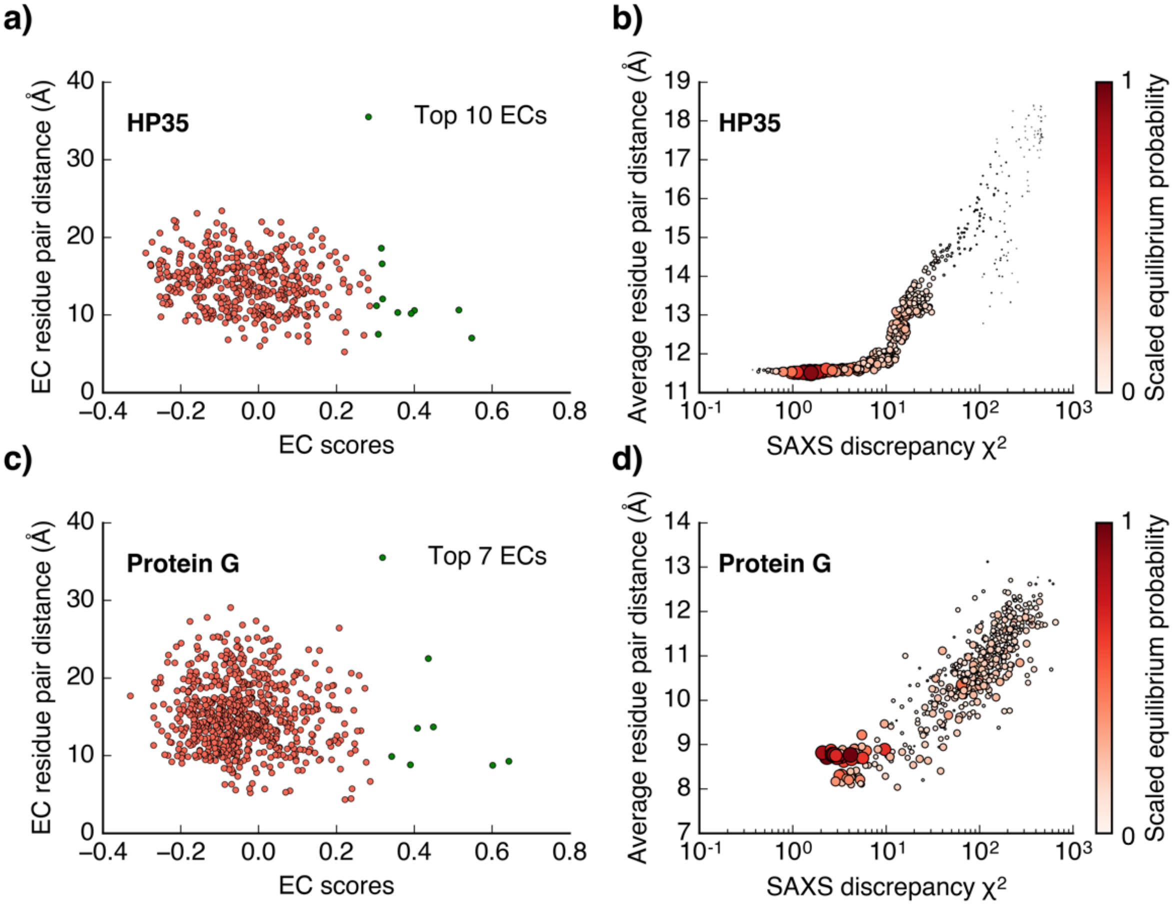

Figure S2. Identification of the ECs for HP35 and Protein G. Distances between evolutionarily coupled residues in (a) the HP35 and (c) the Protein G native folded structures versus evolutionary coupling scores. Couplings with scores above 0.3 were used in structural characterization and adaptive sampling. Average EC residue pair distances of all states versus their SAXS discrepancy scores for the (b) HP35 and (d) Protein G. Free energy, SAXS discrepancy scores, and average EC residue pair distance can be used collectively for identification of native states of proteins.

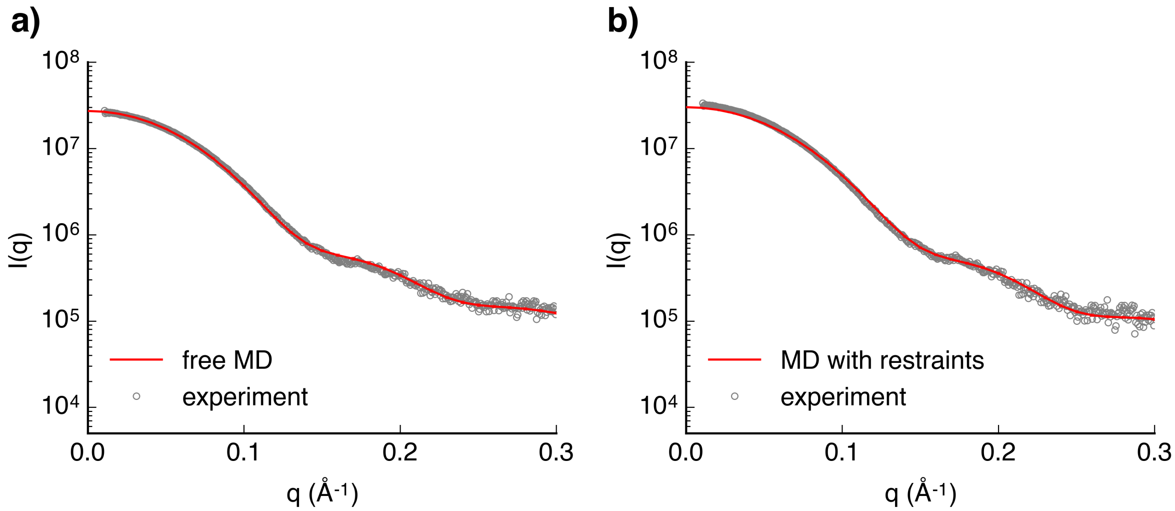

Figure S3. Fitting the PYR1 experimental SAXS data to SAXS profiles calculated from free and constrained MD simulations. The PYR1 experimental SAXS data are fitted to the theoretical SAXS profiles calculated from (a) free MD simulations and (b) MD simulations with restraints on complex backbones, with χ^2 (on logarithmic scale) of 0.006, 0.011 respectively.

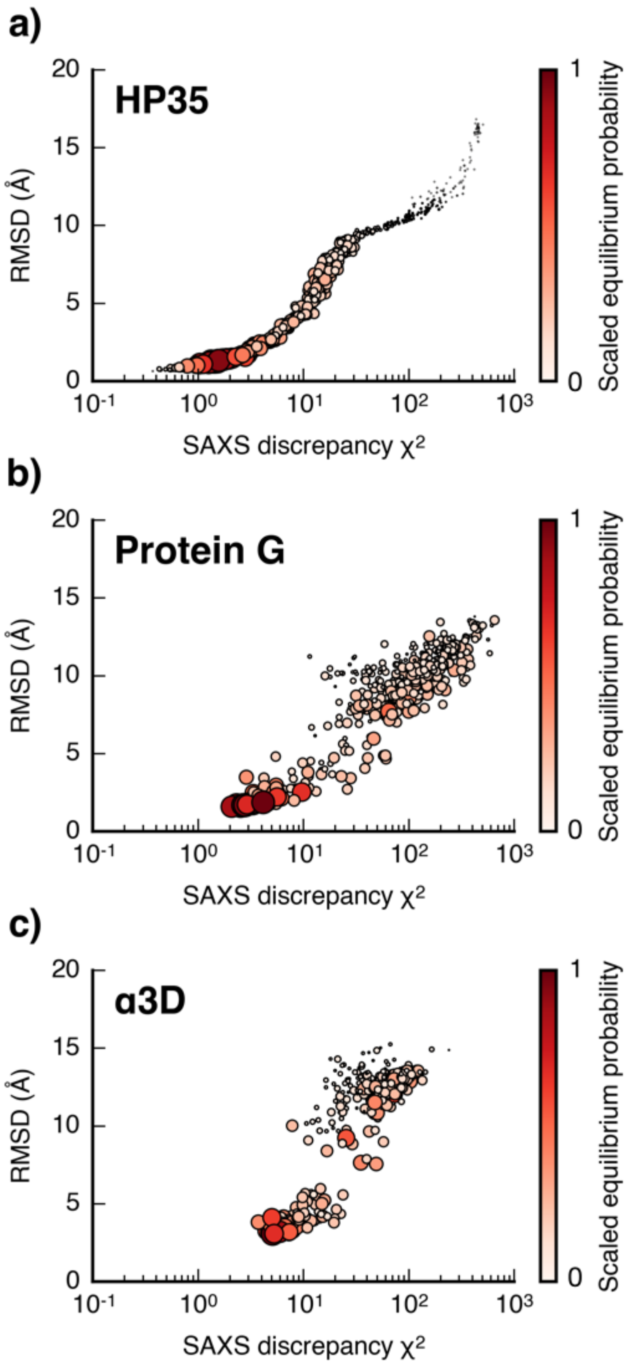

Figure S4. Structure comparison with the crystal structures. The plots of individual MSM state average C_α RMSD from the crystal structure with respect to their average SAXS discrepancy values for the (**a**) HP35, (**b**) protein G, and (**c**) α3D.

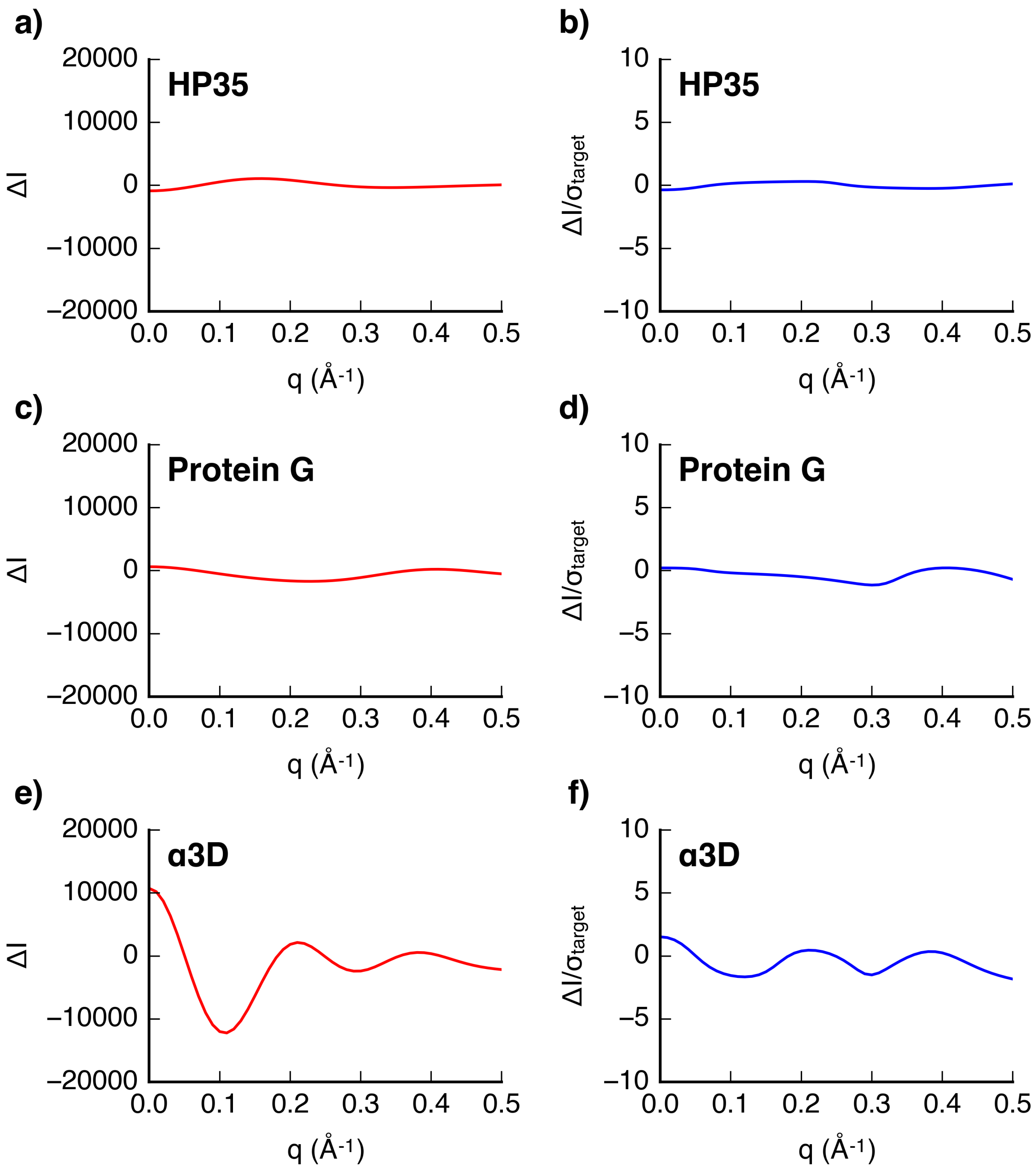

Figure S5. Residuals between the post-MD model SAXS profiles of single domain proteins and the target SAXS profiles. Residuals (a), (c), (e) ΔI and (b), (d), (f) $\Delta I/\sigma_{target}$ normalized by the errors of the target SAXS profiles for HP35, protein G and α3D.

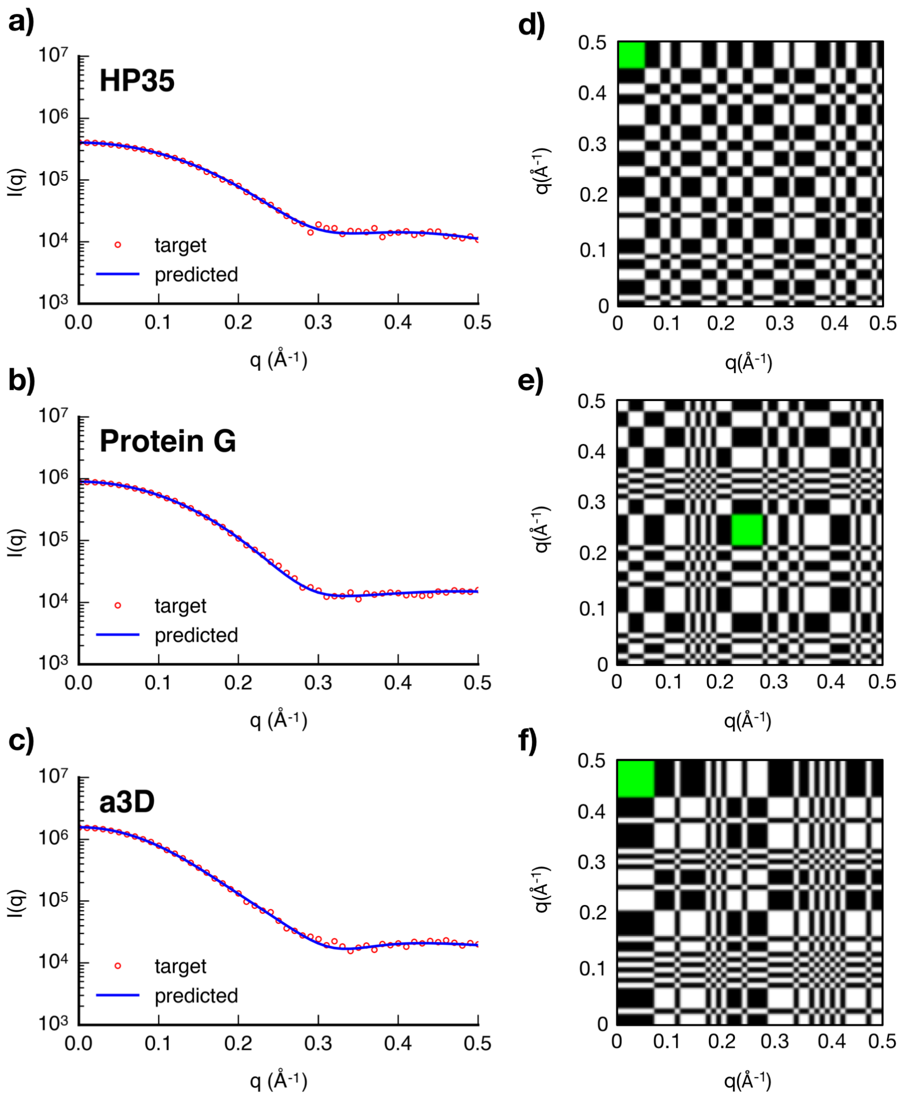

Figure S6. Fitting assessment of post-MD model SAXS profiles of single domain proteins to target SAXS data. Target and predicted SAXS profiles of (a) HP35, (b) protein G, and (**c**) α3D. Gaussian random noises were added to the original calculated scattering intensities $I_{target}(q_i)$ to account for the errors in the target SAXS profiles (see methods). Pairwise comparisons of target and predicted SAXS profiles for (d) HP35, (e) protein G, and (f) α3D. The corresponding probabilities of similarity (p-value) of the two-frame comparisons are 0.8274 (51 points, C=5), 0.2273 (51 points, C=6), and 0.3146 (51 points, C=7). The fitting assessments were carried out in the ATSAS Primus software package.

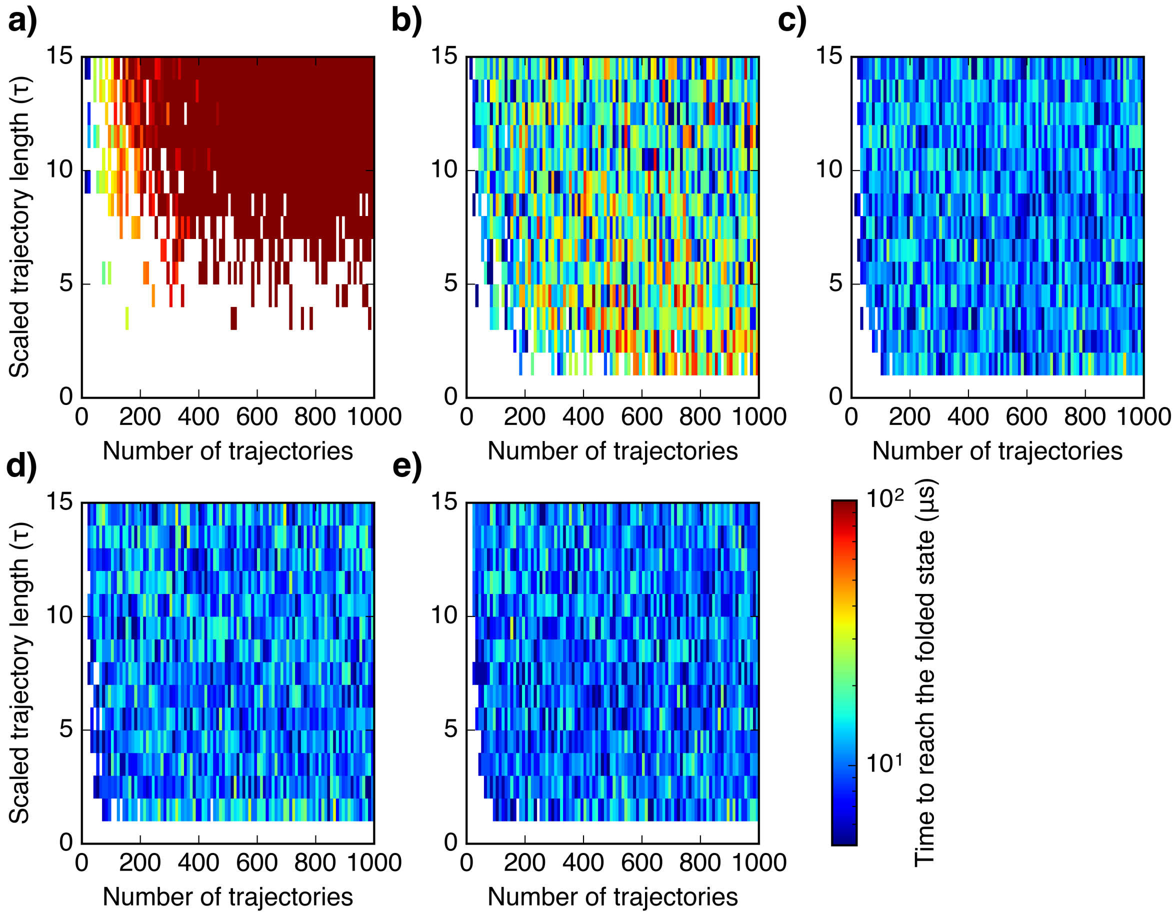

Figure S7. Enhanced efficiency in sampling the folding of Protein G. Total simulation time required to reach the folded state from an arbitrary unfolded state for sets of samplings using (**a**) traditional long simulation, (**b**) random adaptive sampling, (**c**) SAXS-guided adaptive sampling, (**d**) EC-guided adaptive sampling, (**e**) SAXS-EC-guided adaptive sampling. Scaled trajectory length is the length of each individual trajectory in each specific sampling scheme by the lag time τ of the MSM. Number of trajectories is the total number of trajectories run for each sampling scheme, given by the product of the number of parallel trajectories and the number of sampling rounds. The average total required sampling times using the 5 different protocols over 1500 sets of samplings (excluding the sets of sampling that do not reach the target state) are 193.25 μs, 22.24 μs, 11.12 μs, 11.20 μs, and 10.3 μs.

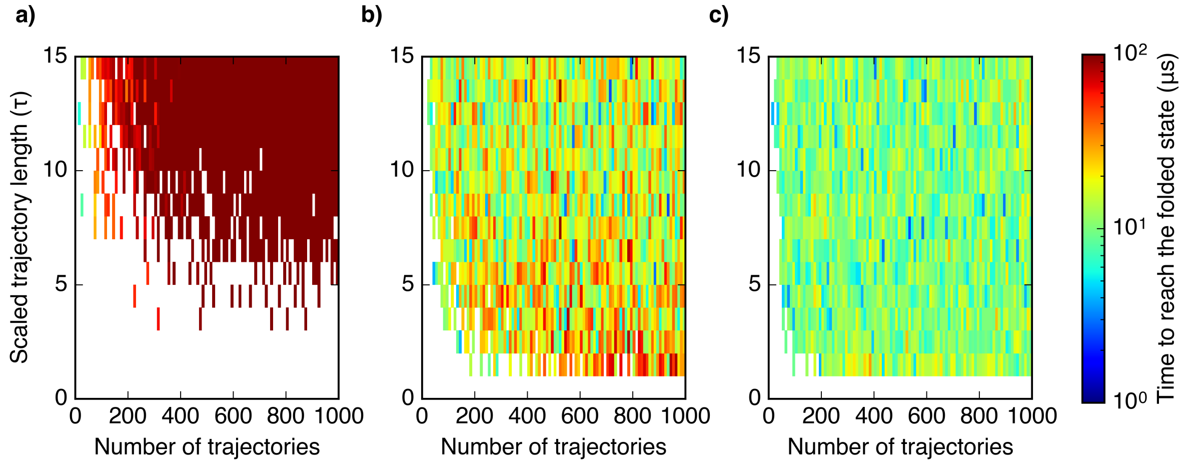

Figure S8. Enhanced efficiency in sampling the folding of α3D. Total simulation time required to reach the folded state from an arbitrary unfolded state for sets of samplings using (**a**) traditional long simulation, (**b**) random adaptive sampling, and (**c**) SAXS-guided adaptive sampling. Scaled trajectory length is the length of each individual trajectory in each specific sampling scheme by the lag time τ of the MSM. Number of trajectories is the total number of trajectories run for each sampling scheme, given by the product of the number of parallel trajectories and the number of sampling rounds. The average total required sampling times using the 3 different protocols over 1500 sets of samplings (excluding the sets of sampling that do not reach the target state) are 202.36 μs, 20.47 μs, and 11.08 μs.

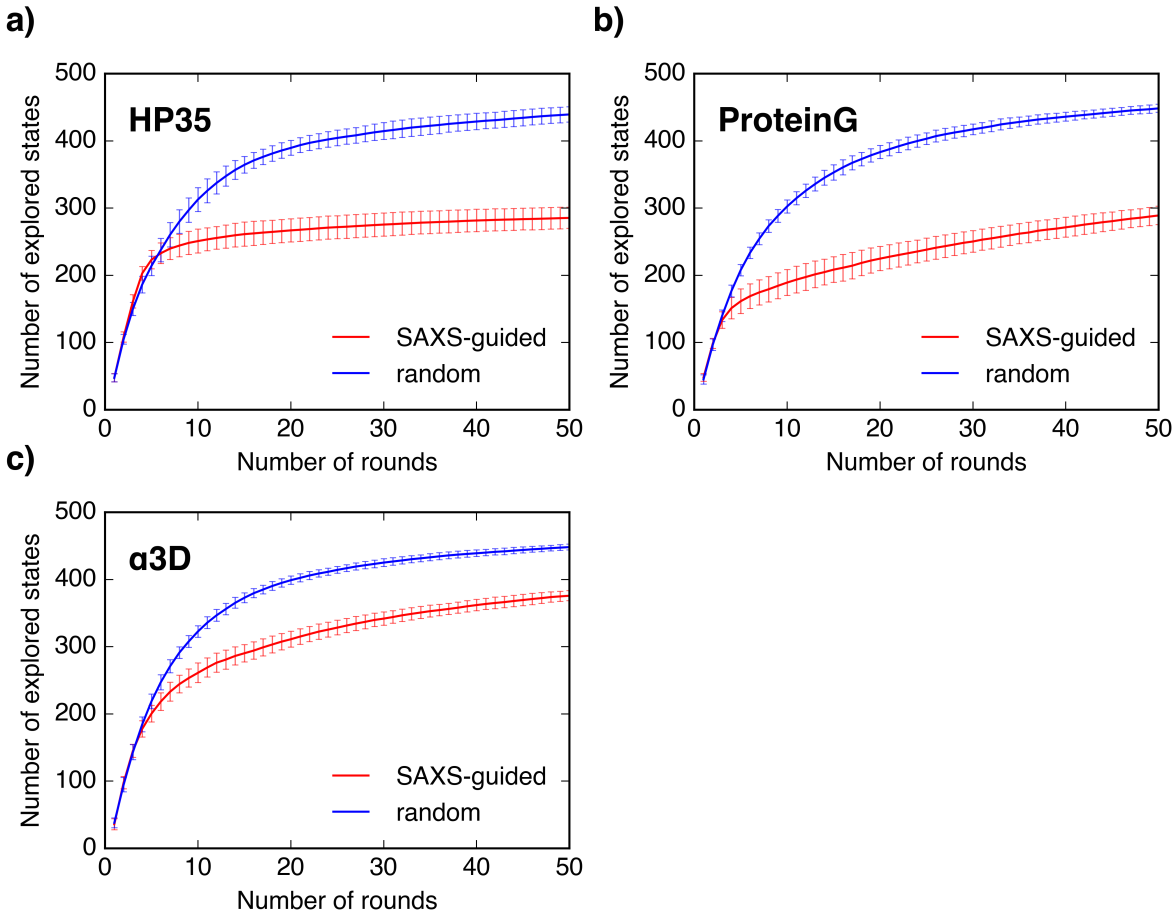

Figure S9. Number of states explored in SAXS-guided and random adaptive sampling. As compared to random adaptive sampling, SAXS-guided adaptive sampling reduces the number of states explored in the sampling of the folding of (a) HP35, (b) Protein G and (c) α3D. The number of explored states are calculated from 50 sets of independent samplings, of which each has 50 rounds, 10 parallel trajectories in each round with length of 10τ.

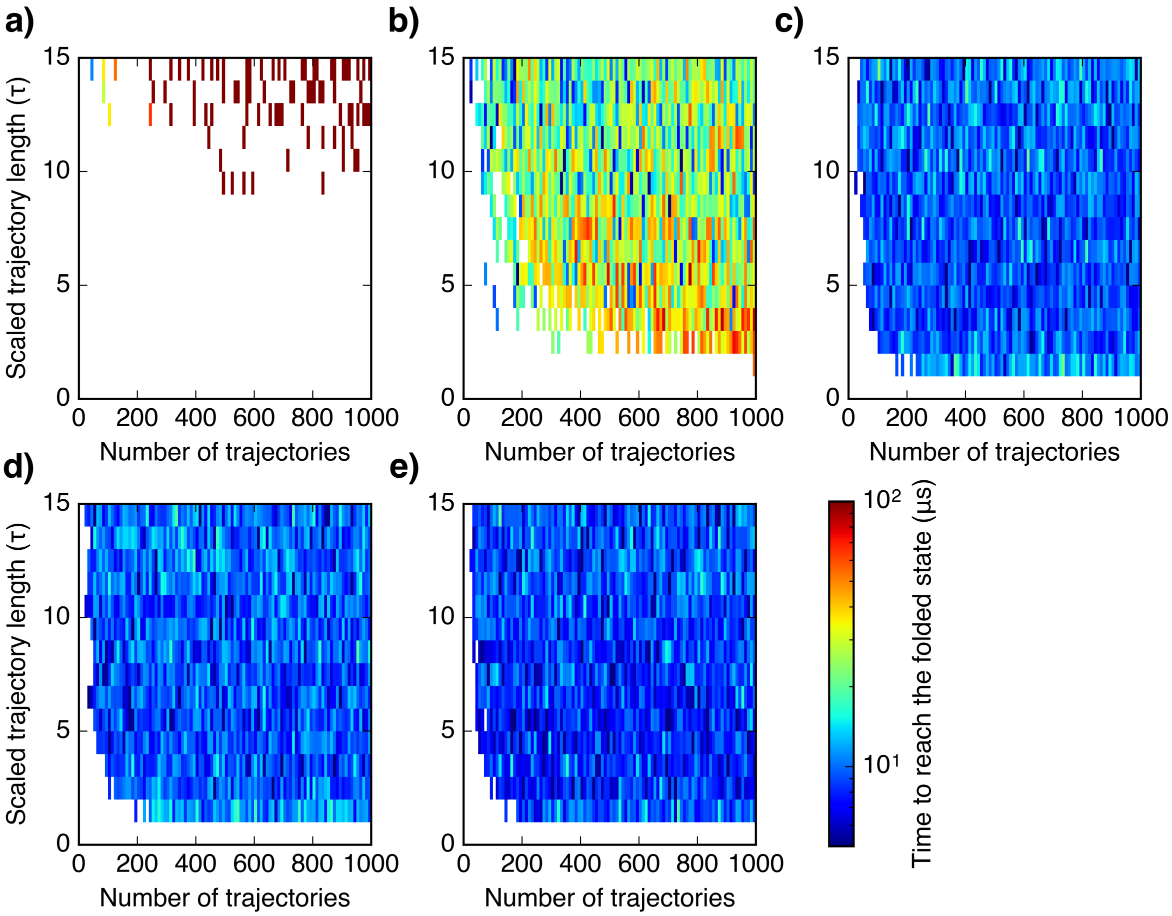

Figure S10. Sampling efficiency comparisons between SAXS-guided, EC-guided and SAXS-EC guided adaptive samplings of the folding of HP35. Total simulation time required to reach the folded state from an arbitrary unfolded state for sets of samplings using (**a**) traditional long simulation, (**b**) random adaptive sampling, (**c**) SAXS-guided adaptive sampling, (**d**) EC-guided adaptive sampling, (**e**) SAXS-EC-guided adaptive sampling. Scaled trajectory length is the length of each individual trajectory in each specific sampling scheme by the lag time τ of the MSM. Number of trajectories is the total number of trajectories run for each sampling scheme, given by the product of the number of parallel trajectories and the number of sampling rounds. The average total required sampling times using the 5 different protocols over 1500 sets of samplings (excluding the sets of sampling that do not reach the target state) are 235.03 μs, 27.61 μs, 9.76 μs, 9.70 μs, and 8.77 μs. The data as shown for **a**-**c** are the same as in Figure 2, which are shown here for comparisons with SAXS-EC-guided adaptive sampling strategy.

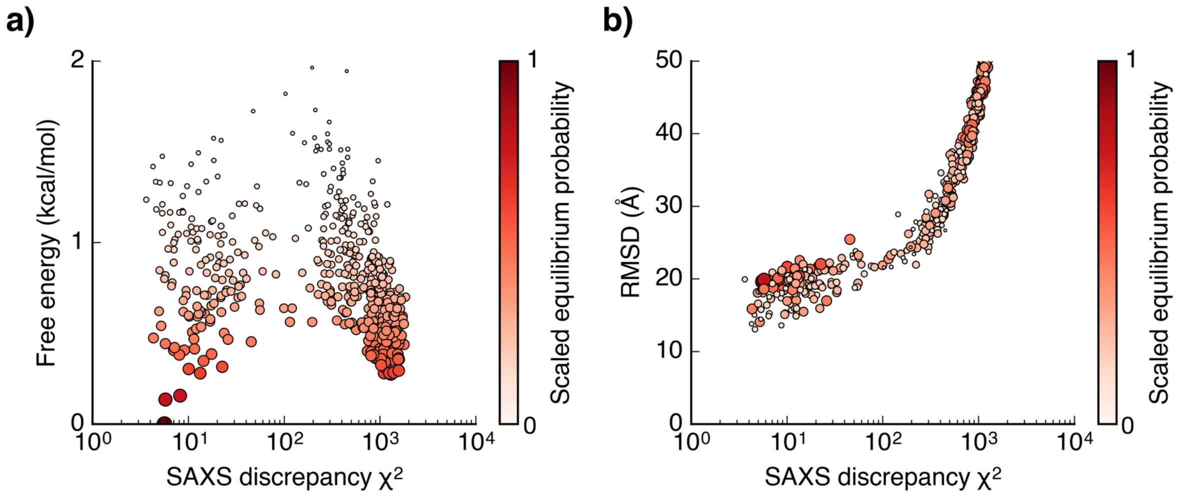

Figure S11. SAXS results for the association of MoaD-MoaE. Plots of (**a**) the free energies and (**b**) the RMSD from the crystal structure with respect to their average SAXS discrepancy values (reduce χ^2).

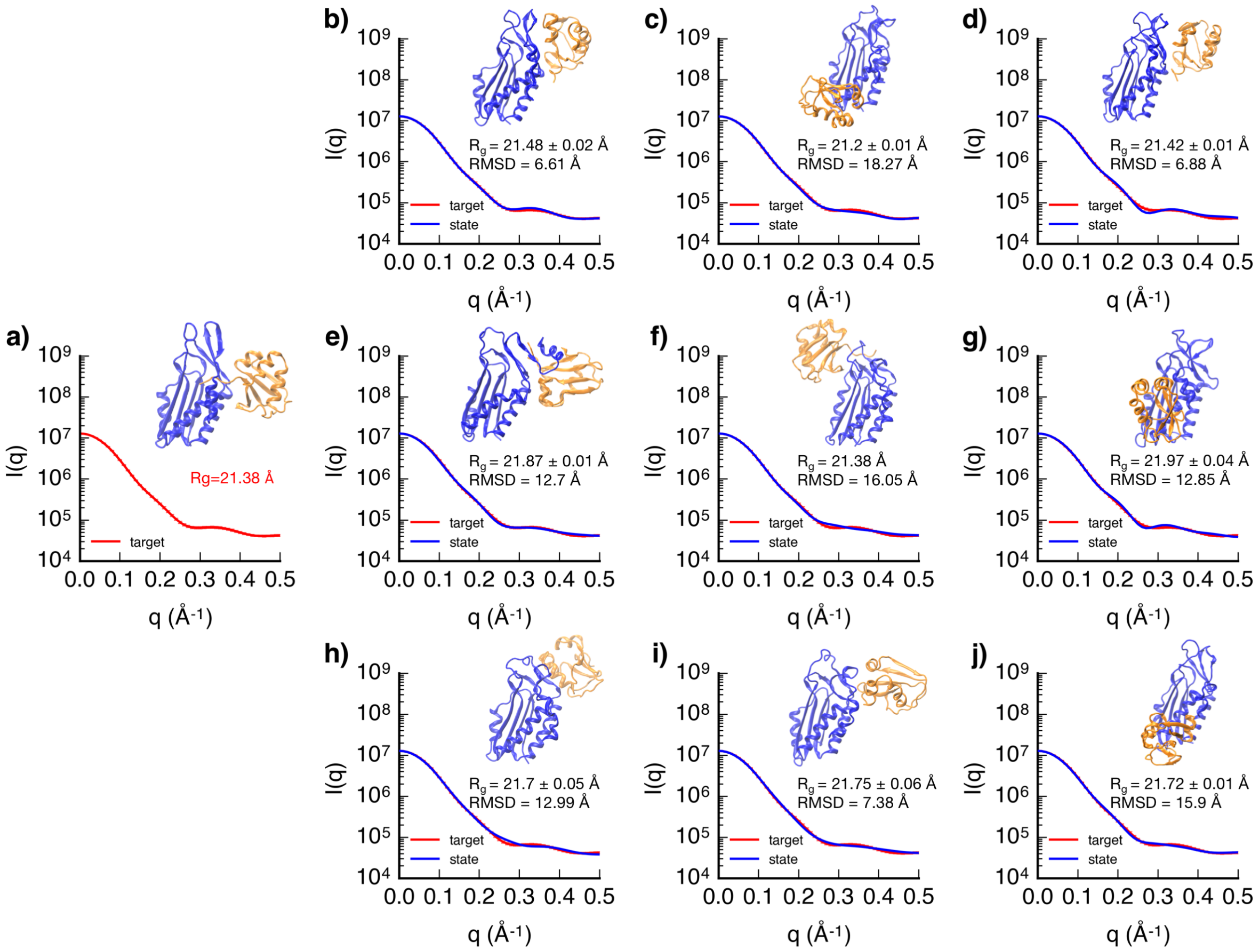

Figure S12. MoaD-MoaE snapshots with the minimal SAXS discrepancy scores. Snapshots and SAXS profiles of (a) the target and (b)-(j) the 9 states with the minimal SAXS discrepancy scores (excluding the predicted state as shown in Figure 3). MoaD and MoaE are colored in orange and blue, respectively. Snapshots are aligned based on the MoaE coordinates.

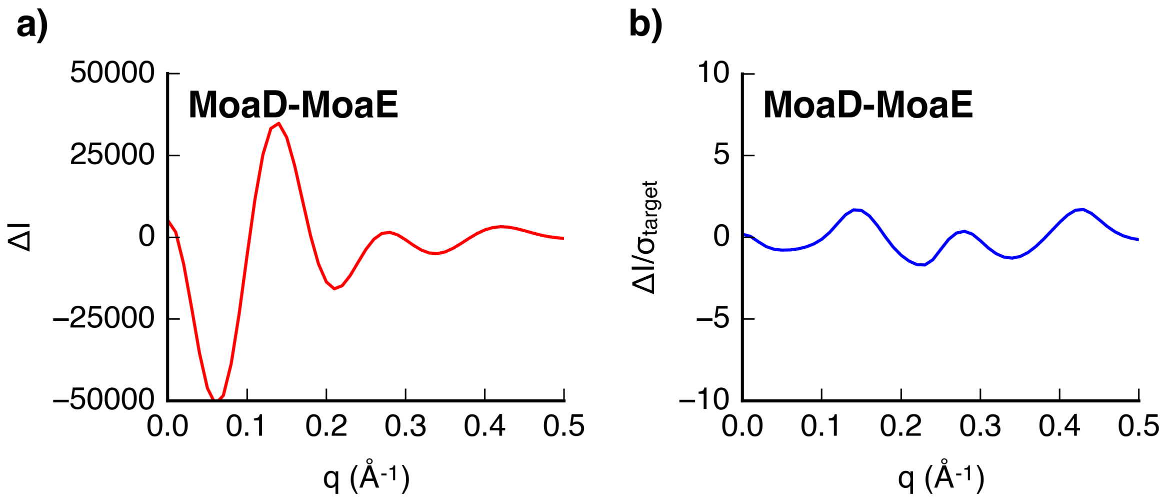

Figure S13. Residuals between the post-MD model SAXS profile of MoaD-MoaE complex and the target SAXS profile. Residuals (a) ΔI and (b) $\Delta I/\sigma_{target}$ normalized by the errors of the target SAXS profile for MoaD-MoaE complex.

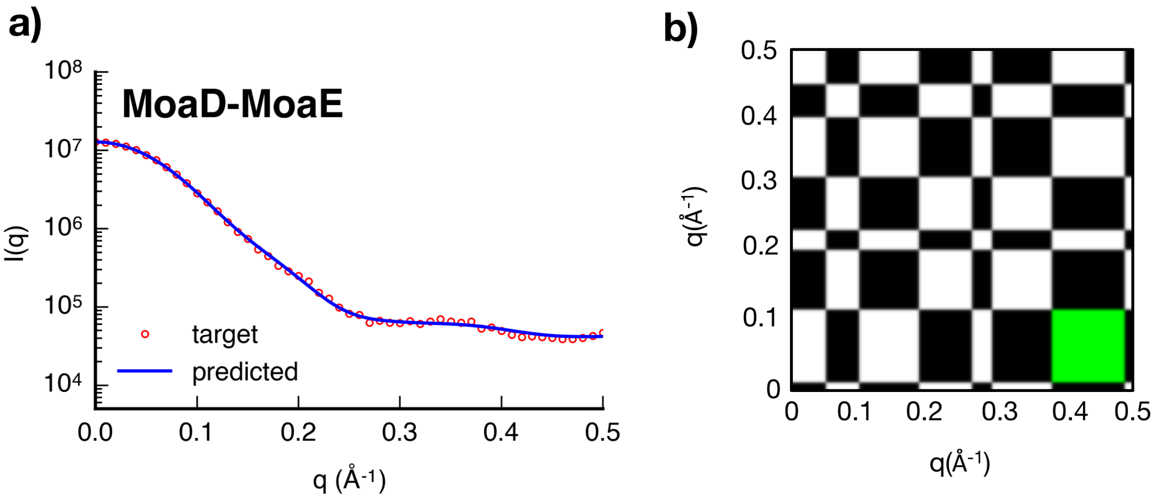

Figure S14. Fitting assessment of post-MD model SAXS profile of MoaD-MoaE complex to target SAXS data. (a) Target and predicted SAXS profiles of MoaD-MoaE. Gaussian random noises were added to the original calculated scattering intensities $I_{target}(q_i)$ to account for the errors in the target SAXS profile (see methods). (b) Pairwise comparisons of target and predicted SAXS profiles for MoaD-MoaE. The corresponding probability of similarity (p-value) of the two-frame comparisons is 0.0836 (51 points, C=9). The fitting assessment was carried out in the ATSAS Primus software package.

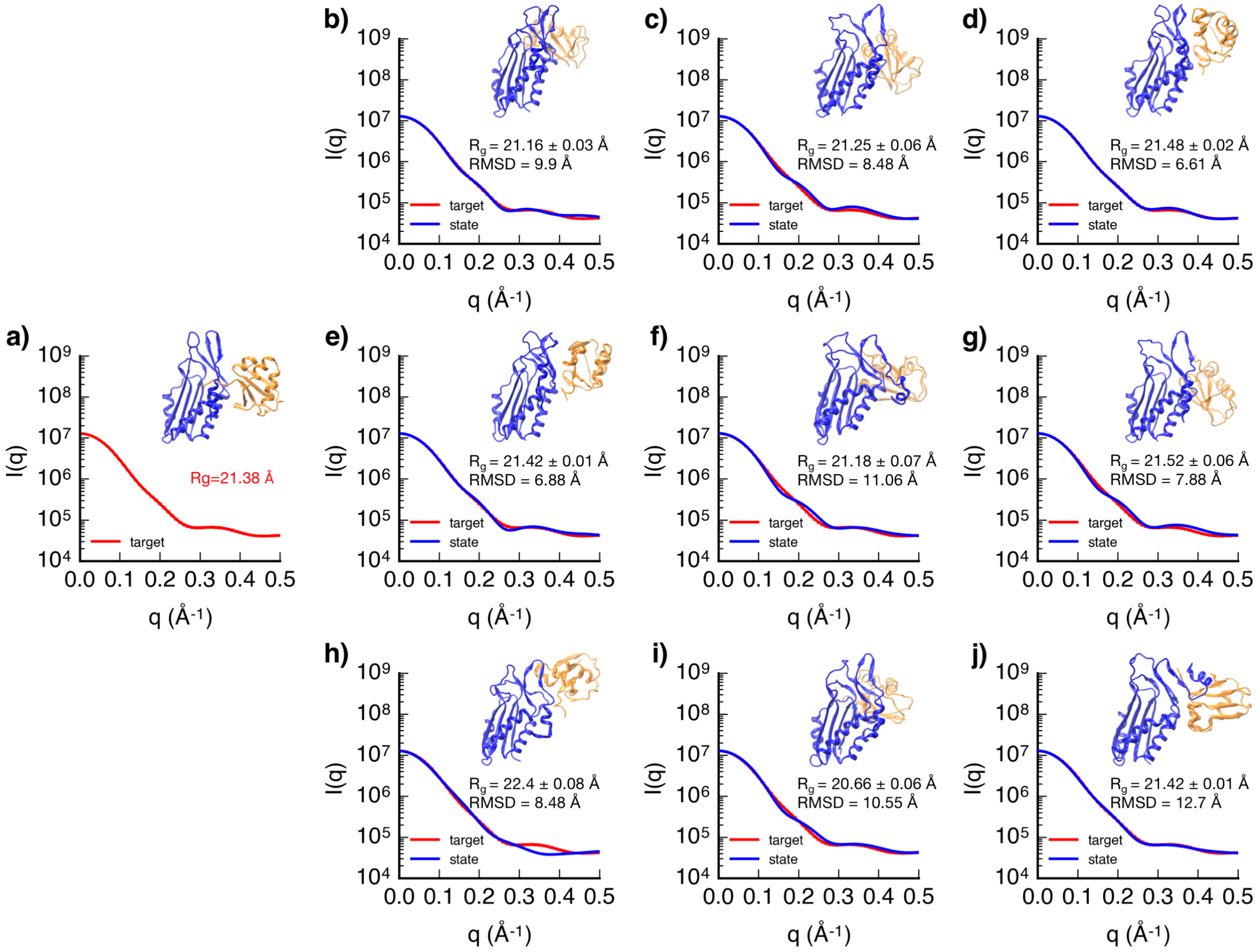

Figure S15. MoaD-MoaE snapshots with the minimal average residue pair distances. Snapshots and SAXS profiles of (a) the target and (b)-(j) 9 states with the minimal average EC residue pair distances (excluding the predicted state as shown in Figure 3). MoaD and MoaE are colored in orange and blue, respectively. Snapshots are aligned based on the MoaE coordinates.

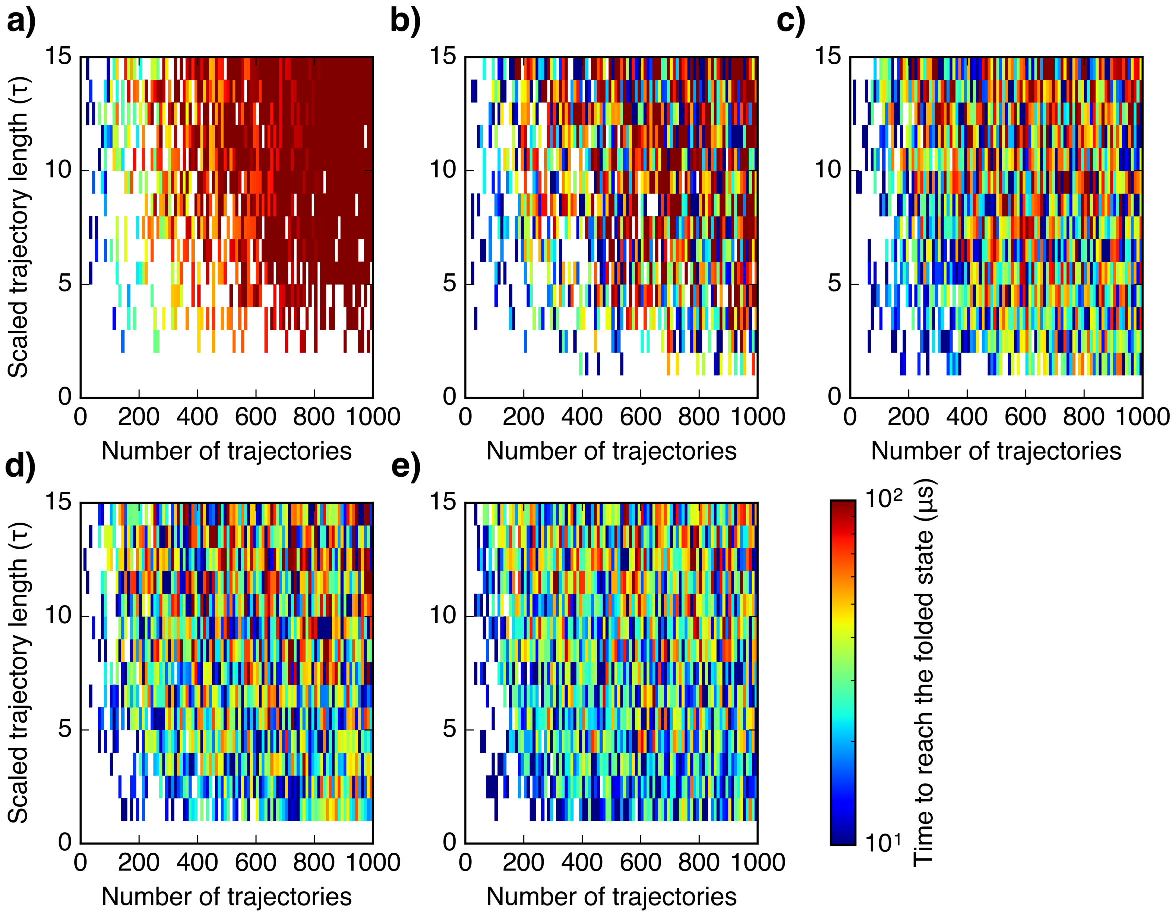

Figure S16. Sampling efficiency comparisons between EC-guided adaptive sampling and SAXS-based adaptive sampling strategies in MoaD-MoaE association. Total simulation time required to reach the folded state from an arbitrary unfolded state for sets of samplings using (**a**) traditional long simulation, (**b**) random adaptive sampling, (**c**) SAXS-guided adaptive sampling, (**d**) EC-guided adaptive sampling, (**e**) SAXS-EC-guided adaptive sampling. Scaled trajectory length is the length of each individual trajectory in each specific sampling scheme by the lag time τ of the MSM. Number of trajectories is the total number of trajectories run for each sampling scheme, given by the product of the number of parallel trajectories and the number of sampling rounds. The average total required sampling times using the 5 different protocols over 1500 sets of samplings (excluding the sets of sampling that do not reach the target state) are 113.28 μs, 61.16 μs, 41.57 μs, 36.35 μs, and 30.64 μs. The data as shown for **a**-**c** and **e** are the same as in Figure 4, which are shown here for comparisons with EC-guided adaptive sampling strategy.

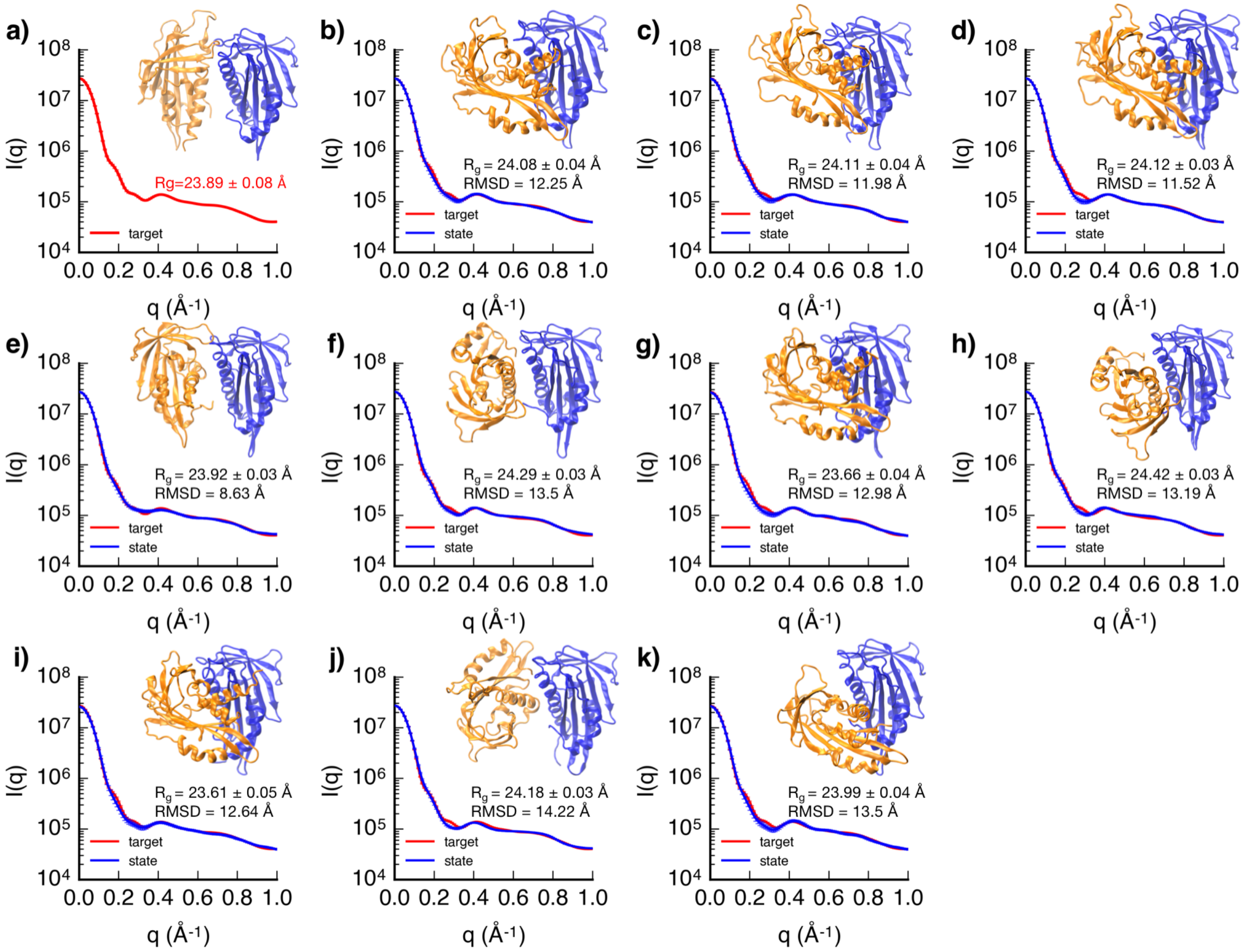

Figure S17. PYR1 snapshots with the minimal SAXS discrepancy scores. Snapshots and SAXS profiles of (a) the target and (b)-(k) 10 states with the minimal SAXS discrepancy scores. Two monomers are colored in orange and blue, respectively. Snapshots are aligned based on the monomer colored in blue.

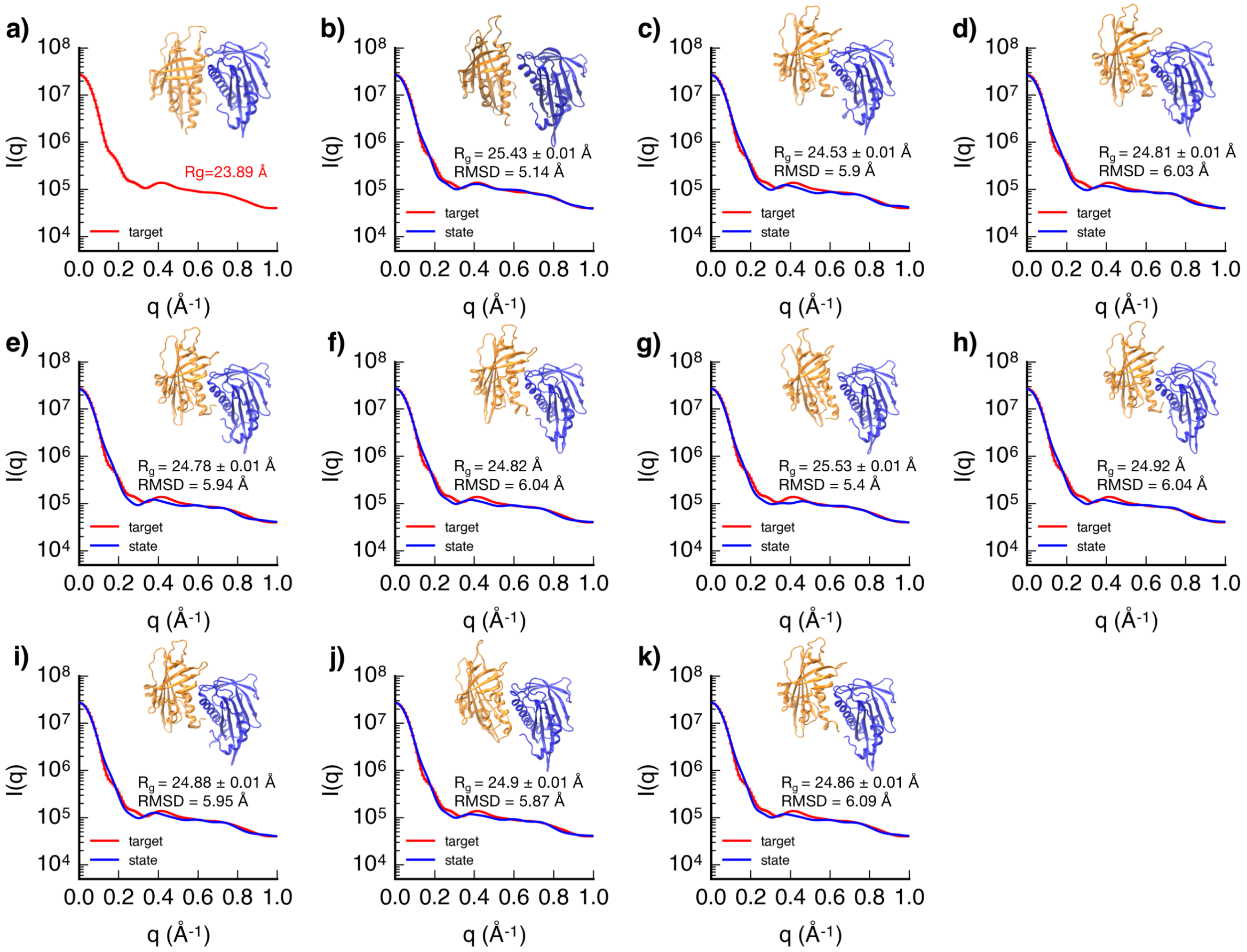

Figure S18. PYR1 snapshots with the minimal average residue pair distances. Snapshots and SAXS profiles of (a) the target and (b)-(k) the 10 states with the minimal average residue pair distances. Two monomers are colored in orange and blue, respectively. Snapshots are aligned based on the monomer colored in blue.

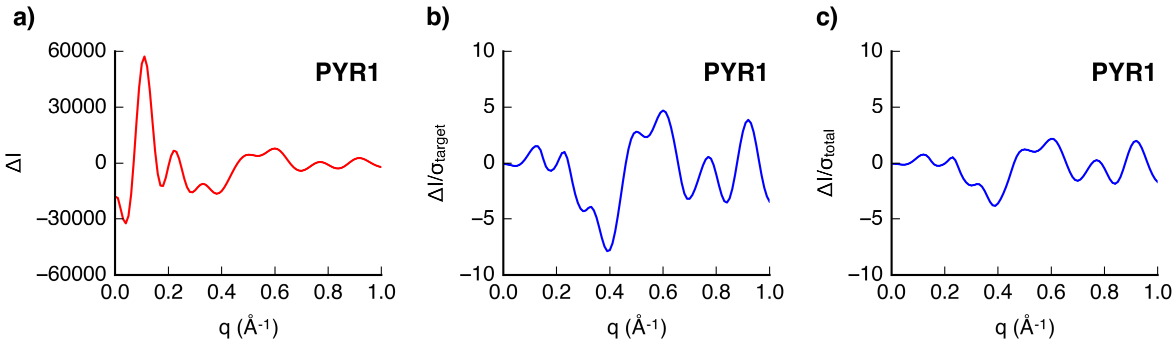

Figure S19. Residuals between the post-MD model SAXS profile of PYR1 complex and the target SAXS profile. Residuals (a) ΔI and (b) $\Delta I/\sigma_{target}$ normalized by the errors of the target SAXS profile and (c) $\Delta I/\sigma_{total}$ normalized by the root of sum of square errors of the target and the predicted SAXS profiles (σ_{total}=$\sqrt{\sigma^2_{target} + \sigma^2_{predicted}}$) for the PYR1 complex.

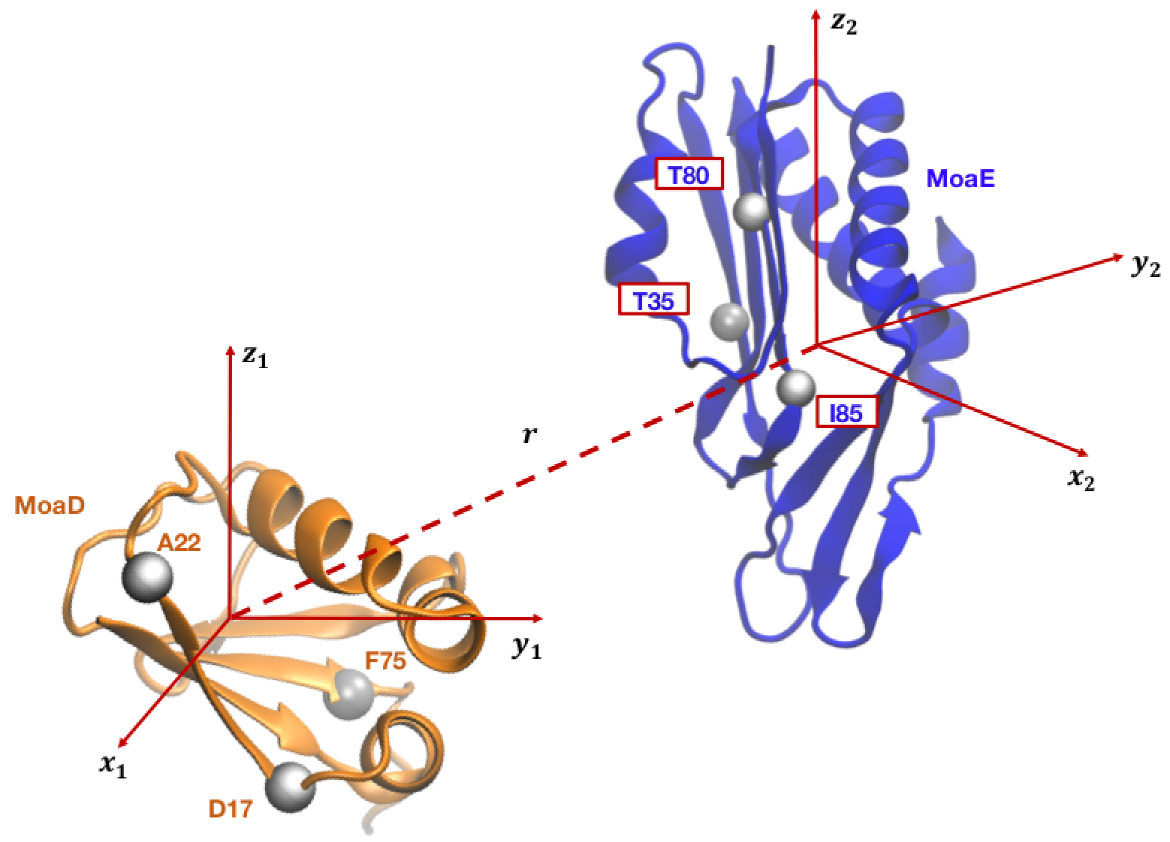

Figure S20. Coordinate systems for clustering the association of MoaD and MoaE. $\boldsymbol{x}_1$ is defined by $\frac{\boldsymbol{r}_{A22}-\boldsymbol{r}_{D17}}{|\boldsymbol{r}_{A22}-\boldsymbol{r}_{D17}|}$, $\boldsymbol{y}_1$ is a unit vector orthogonal to $\boldsymbol{x}_1$ in the plane defined by D17, A22 and F75 of MoaD. $\boldsymbol{y}_1=\frac{(\boldsymbol{r}_{F75}-\boldsymbol{r}_{D17})-[(\boldsymbol{r}_{F75}-\boldsymbol{r}_{D17})\cdot\boldsymbol{x}_1]\boldsymbol{x}_1}{|(\boldsymbol{r}_{F75}-\boldsymbol{r}_{D17})-[(\boldsymbol{r}_{F75}-\boldsymbol{r}_{D17})\cdot\boldsymbol{x}_1]\boldsymbol{x}_1|}$. z_1 is then defined by $\frac{\boldsymbol{x}_1\times\boldsymbol{y}_1}{|\boldsymbol{x}_1\times\boldsymbol{y}_1|}$. Similarly, $\boldsymbol{x}_2$, $\boldsymbol{y}_2$, z_2 were defined by T35, T80 and I85 of MoaE.
